# RAP2.4a Is Transported through the Phloem to Regulate Cold and Heat Tolerance in Papaya Tree (*Carica papaya* cv. Maradol): Implications for Protection Against Abiotic Stress

**DOI:** 10.1371/journal.pone.0165030

**Published:** 2016-10-20

**Authors:** Luis Figueroa-Yañez, Alejandro Pereira-Santana, Ana Arroyo-Herrera, Ulises Rodriguez-Corona, Felipe Sanchez-Teyer, Jorge Espadas-Alcocer, Francisco Espadas-Gil, Felipe Barredo-Pool, Enrique Castaño, Luis Carlos Rodriguez-Zapata

**Affiliations:** 1 Unidad de Biotecnología, Centro de Investigación Científica de Yucatán, Mérida, Yucatán, México; 2 Unidad de Bioquímica y Biología Molecular de Plantas, Centro de Investigación Científica de Yucatán, Mérida, Yucatán, México; 3 Laboratorio de Farmacología, Facultad de Química, Universidad Autónoma de Yucatán, Mérida, Yucatán, México; Bhabha Atomic Research Centre, INDIA

## Abstract

Plants respond to stress through metabolic and morphological changes that increase their ability to survive and grow. To this end, several transcription factor families are responsible for transmitting the signals that are required for these changes. Here, we studied the transcription factor superfamily AP2/ERF, particularly, RAP2.4 from *Carica papaya* cv. Maradol. We isolated four genes (*CpRap2*.*4a*, *CpRAap2*.*4b*, *CpRap2*.*1* and *CpRap2*.*10*), and an *in silico* analysis showed that the four genes encode proteins that contain a conserved APETALA2 (AP2) domain located within group I and II transcription factors of the AP2/ERF superfamily. Semiquantitative PCR experiments indicated that each Cp*Rap2* gene is differentially expressed under stress conditions, such as extreme temperatures. Moreover, genetic transformants of tobacco plants overexpressing *CpRap2*.*4a* and *CpRap2*.*4b* genes show a high level of tolerance to cold and heat stress compared to non-transformed plants. Confocal microscopy analysis of tobacco transgenic plants showed that CpRAP2.4a and CpRAP2.4b proteins were mainly localized to the nuclei of cells from the leaves and roots and also in the sieve elements. Moreover, the movement of CpRap2.4a RNA in tobacco grafting was analyzed. Our results indicate that *CpRap2*.*4a* and *CpRap2*.*4b* RNA in the papaya tree have a functional role in the response to stress conditions such as exposure to extreme temperatures via direct translation outside the parental RNA cell.

## Introduction

The major adverse environmental factors that can affect plant growth and crop production are drought, cold and high salinity. Under these stress conditions, a variety of genes are induced, which enable plants to respond to these abiotic stressors. There are several transcription regulatory networks involved in stress-induced changes in gene expression [1]. Stress-induced genes can up-regulate the expression of many downstream genes that provide abiotic stress tolerance to extreme temperatures, drought, and high salinity [2, 3]. Some of these transcription factors that are involved in abiotic stress tolerance belong to the APETALA2 superfamily (AP2)/ethylene-responsive element-binding (ERF). The AP2/ERF transcription factors (TFs) bind to *cis*-DRE/CTR (A/GCCGAC) sites located in specific regions of a promoter to regulate the transcriptional expression of different stress-related genes. The AP2/ERF TF superfamily hosts a highly conserved AP2 domain, which consists of approximately 60 to 70 amino acids and is involved in DNA binding [4]. The Arabidopsis AP2/ERF genes function as key developmental regulators or important mediators of responses to various environmental stress signals, such as cold, salt, and drought [5, 6]. A role for AP2/ERF genes in light signaling regulation is suggested by the observation that several Arabidopsis AP2/ERF transcripts, including RAP2.4 (At1g78080), are significantly and rapidly regulated by light [7–11]. In *Arabidopsis thaliana*, overexpression or mutation of the *AtRap2*.*4* gene causes altered expression of representative light- and ethylene-responsive genes, causing different defects in multiple developmental processes that are regulated by light and ethylene, including hypocotyl elongation and gravitropism, apical hook formation and cotyledon expansion, flowering time, root elongation, root hair formation, and drought tolerance [12]. On the other hand, RAP2.4a from *A*. *thaliana* controls the transcript abundance of prominent chloroplast antioxidant enzymes through binding to the CGCG core of a CE3-like element. These findings suggest that the redox sensitivity of RAP2.4a establishes an efficient switch mechanism for redox control of the nuclear gene activity of chloroplast antioxidants, of which RAP2.4 is a redox-sensor and a transducer of redox information [13]. Many RAP2.4 transcription factors have been isolated from several plants, and their involvement in stress tolerance has been proposed. In soybean, early perception of water deprivation was critical for the recruitment of genes that promote plant tolerance. In addition to RAP2.4, the transcript levels of nine genes were differentially expressed in response to drought stress [14]. Similar results were obtained when apical shoots of cassava were subjected to cold stress of 7°C. A total of 508 transcripts were identified as early cold-responsive genes, and only 319 sequences had functional descriptions compared with Arabidopsis proteins. Various stress-related genes with a wide range of biological functions were found, including signal transduction components (e.g., MAP kinase 4), transcription factors (e.g., RAP2.4 and RAP2.11), and reactive oxygen species (ROS) scavenging enzymes (e.g., catalase 2), as well as photosynthesis-related genes, e.g., PsaL [15].

The plant vascular system serves as the main route for the long- and short-distance transport of various compounds throughout the plant body, and serves as a means of long-distance communication. The vascular system consists of two major tissues types, xylem, which conducts water and nutrients, and phloem, which transport mainly organic compounds. Little information has been generated on the roles of the different proteins and RNA species that are involved in the abiotic stress response, or on how these molecules communicate over long and short distances (see the review of Ursache, Heo [16]); however, there is evidence that some proteins and microRNAs are present in the phloem and that these molecules participate in the response to different abiotic stressors (see the review of Kehr and Buhtz [17]). Zhang, Yang [18] demonstrated that the *Athspr* promoter from Arabidopsis has vascular tissue-specific activity and that *Athspr* has multiple functions in vascular development, as it contains regulatory elements that respond to phytohormones, light, biotic and abiotic stresses, as well as those regulating tissue-specific expression. Also in transgenic rice plants overexpressing promoter *TdPip2*,*1* in fusion with the *TdPip2*,*1* gene from Durum wheat showed enhanced drought tolerance, while wild-type plants were more sensitive and exhibited symptoms of wilting and chlorosis. Histological sections revealed the accumulation of GUS products in the phloem and xylem, and in some cells adjacent to the xylem [19]. Szabala, Fudali [20] found that SK3 dehydrin DHn24 from *Solanum sogarandinum* was specifically localized to sieve elements (ses) and companion cells (ccs) of roots and stems in cold-acclimated plants. They determined that homologous DHn24 proteins in *Capsicum annuum* and *Lycopersicon chilense* were constitutively expressed, but their protein levels were upregulated preferentially during drought stress. On the other hand, a transcriptome analysis of phloem sap from *Fraxinus spp*. was performed, and several AP2 sequences were found [21]. The functions of most of the genes in sap are unknown, thus open questions remain, for example, whether these genes are involved in a specific biological process, such as the abiotic stress response, and the roles of these RNA transcripts in the sap.

Furthermore there is no evidence that RAP2.4 TFs respond to abiotic stress in a fruit tree model. The yield of papaya, a fruit tree crop known for its nutritional benefits and medicinal applications, is severely affected by different types of abiotic stress, such as drought, extreme temperature, and salinity. To understand the function of the RAP2 genes under abiotic stress, more specifically cold and heat stress, we isolated and characterized four RAP2 genes from *C*. *papaya* cv. Maradol, namely, *CpRap2*.*4a*, *CpRap2*.*4b*, *CpRap2*.*1*, and *CpRap2*.*10*. We identified the tissues and cells where RAP2 proteins are expressed. In addition, we investigated the role of *Rap2*.*4a* mRNA in long-distance communication in the papaya tree by grafting with tobacco plants. The transgenic RAP2.4a graft modified the physiological response to stress of the wild-type scion.

## Materials and Methods

### AP2/ERF gene family searches and retrieval

A Hidden Markov Model (HMM) profile was constructed for the identification of new AP2/ERF genes. To build this profile, 595 AP2/ERF genes from six different species (*C*. *reinhardtii*, *P*. *patens*, *S*. *moellendorffii*, *O*. *sativa* subsp. Japonica, *V*. *vinifera*, and *A*. *thaliana*) were retrieved from The Plant Transcription Factor Database v3.0 [22]. A multiple sequence alignment was performed with Clustal Omega [23], and then the alignment was manually curated. The HMM model was constructed using the HMMER software package v3.1 [24], curated with the cut-off values and used to detect AP2/ERF genes in seven plant species and a green alga. The gene model of *Arabidopsis thaliana* was downloaded from the TAIR database [25]. The gene files of *Carica papaya* (ASGPBv0.4), *Chlamydomonas reinhardtii* (v5.5), *Physcomitrella patens* (v3.0), *Selaginella moellendorffii* (v1.0), and *Solanum lycopersicum* (iTAG2.3) were downloaded from Phytozome v9 [26]. The gene annotation of *Musa acuminata* (DH-Pahang, CIRAD) and *Oryza sativa* subsp. Japonica (IRGSP-1.0) were downloaded from EnsemblPlants (http://plants.ensembl.org/index.html, EMBL-EBI).

### Sequence analysis and evolutionary reconstruction

The 832 retrieved amino acid sequences were subjected to a motif scan using the Pfam database v27.0 [27] to confirm the presence of the AP2/ERF domain. Then all the sequences were clustered using CD-HIT [28] with an identity cut-off of 0.9 to exclude isoforms and duplicated genes. Multiple sequence alignments of all of the AP2/ERF selected proteins were performed using Muscle [29]. The alignments were tested with the statistical package ProtTest 2.4 [30] to find the best evolutionary model to use in a maximum likelihood (ML) analysis. The phylogenetic reconstructions were carried out using the RAxML suite v.8.0.26 [31] with a LG + Γ substitution model and 1,000 bootstrap replicates. The AP2/ERF sequences of *C*. *reinhardtii* were used as the outgroup, based on the parsimony-based rooting [32, 33]. Groups formed in the phylogenetic analysis were named according to a previous study [6]. 27 sequences of the group I subgroup of RAP2.4 and 33 sequences of the group II subgroup of RAP2.1/2.9/2.10 were taken for a ML tree. The topology for the best-scoring trees was visualized using Figtree v1.4 (http://www.molecularevolution.org). The MEME software [34] was used to identify conserved motifs in the AP2/ERF designated clades based on the occurrence parameter for a single motif “one per sequence” and the maximum number of sites of each motif. The amino acid sequence similarities were obtained using Muscle [29] and visualized using BOXSHADE v3.31C (http://boxshade.sourceforge.net/).

### Accession numbers

The sequence data from the genes isolated from *C*. *papaya* are available from the GenBank database under the following accession numbers: CpRAP2.4a (KU065114), CpRAP2.4b (KU065115), CpRAP2.1 (KU065116), and CpRAP2.10 (KU065117), CpDREB1A (KU065118), and CpHSP70 (KU065119).

### Plants, growth conditions and stress treatments in papaya

Seeds from papaya (*Carica papaya* cv. Maradol) were surface-sterilized using 1.05% sodium hypochlorite and 200 μl of Tween-20 for one hour at 27°C. Subsequently, the papaya's seeds were shaken in 50 ml of 1.0-M KNO3 for 24 h at 27°C [35]. Floating seeds were discarded, and submerged seeds were shaken in 100 ml of sterile water at 32°C for three days or until the testae cracked [36]. Once the seeds germinated, seedlings were planted in a potting mixture of Horti Pearl, Sunshine, Vermiculite, and Peat moss (2:2:2:1). 180 plants were grown under a photoperiod of 16 h light/8 h dark at 25°C for six weeks. For the temperature stress treatments, three papaya plants from four different lines were selected based on size and health similarity prior the experiment to maintain a uniform start point. The plants were then incubated at either 4°C or 40°C for different exposure times.

### Nucleic acid extraction

Total RNA from different plant tissues was isolated using the TRIzol Reagent (Ambion, http://www.ambion.com) according to the manufacturer´s instructions. Sap extraction was performed by transverse cutting of the stem of papaya seedlings, and the fluid sap was taken with a tip pre-loaded with 20 μl of TRIzol Reagent, avoiding the plant stem edges. For total DNA extraction, 5 g of leaf tissue was ground and mixed with 15 ml of extraction buffer [0.1-M Tris-HCl, pH 8.0; 1.0-M NaCl; 0.02-M EDTA, pH 8.0; 2% (w/v) CTAB; 2% (w/v) polyvinlypyrrolidone-40; 0.2% (v/v) 2-mercaptoethanol], and incubated for 1 h at 70°C. The solution was then twice extracted with equal volumes of chloroform:isoamyl alcohol (CIA, 24:1, v/v) and centrifuged at 15,000 × g, 4°C for 10 min. The aqueous phase was transferred to a 1.5 ml tube, and the DNA was precipitated with an equal volume of isopropanol, and kept at -80°C for 1 h. The DNA was then spooled out and washed with 80% ethanol-15-mM ammonium acetate, and a second wash was performed with 100% ethanol. Each washing step was performed for 20 min with gentle shaking. The DNA pellet was air dried and dissolved in 100 μl of Tris-EDTA buffer (10-mM Tris, pH 8.0; 1-mM EDTA, pH 8.0; 1-M NaCl) by incubating at 65°C for 1 h.

### cDNA synthesis and semiquantitative reverse transcription PCR (RT-PCR)

For semi-quantitative RT-PCR, 1 μg of total RNA was used for the cDNA synthesis. Reverse transcription was performed by SMARTerTM PCR cDNA Synthesis Kit (Clontech, http://www.clontech.com), according to the manufacturer’s instructions. Each cDNA was diluted 1:5, and 2 μl of diluted sample was used in a 30 cycle PCR program with gene-specific primers as follows: The primers used: CpRap2.4a (forward) 5´- ATG CCT CAA CCT ATT TCA AAC GC-3´ and (reverse) 5´- TCA TGA CAA TAT GGA GGC CCA AT-3´); CpRap2.4b (forward) 5´- ATG GCA ACA GCT ATA GAT-3´ and (reverse) 5´- TTA ATA TTC GGA TAG TTT CCT TAT-3´); CpRap2.1 (forward) 5´- ATG GAA GGA GAG TGT TGT TCG ACG-3´ and (reverse) 5´- TCA GTC TTC ATC GGA AGT TTC CGG-3´; and CpRap2.10 (forward) 5´- ATG GAG GGT GGA GCG GAG-3´ and (reverse) 5´- TCA CTC GCC ATC CGA ACT ATC TG-3´). The housekeeping 18S gene was amplified from each sample to normalize the level of each test gene using 18S universal primers (forward, 5´-CGG CTA CCA CAT CCA AGG AA-3 and reverse, 5´-GCT GGA ATT ACC GCG GCT-3´). PCR products were run on a 2% agarose gel with ethidium bromide and visualized on a Molecular Imager® Gel Doc™ XR+ System.

### Plasmid construction for plant transformation

PCR products of *CpRap2*.*4a*, *CpRap2*.*4b*, *CpRap2*.*1*, and *CpRap2*.*10* were purified using a QIAprep Spin Miniprep Kit (QUIAGEN, https://www.qiagen.com) and then cloned into the pGem-T easy vector system (Promega, https://www.promega.com) according to the manufacturer's instructions. The ligations were transformed into *E*. *coli* XL1-Blue competent cells using a calcium protocol transformation [37], and sequenced by Sanger (Cinvestav-Langebio, Irapuato, Mexico). Subcloning of *CpRap2*.*4a*, *CpRap2*.*4b*, *CpRap2*.*1* and *CpRap2*.*10* were performed by using the following primers: CpRap2.4a (forward) 5´-AAA AAG CAG GCT TCA CCA TGC CTC AAC CTA TTT CAA ACG CG-3´ and (reverse) 5´-AGA AAG CTG GGT GTG ACA ATA TGG AGG CCC AAT CGA T-3´; CpRap2.4b (forward) 5´-AAA AAG CAG GCT TCA CCA TGG CAA CAG CTA TAG AT-3´ and (reverse) 5´-AGA AAG CTG GGT GAT ATT CGG ATA GTT TCC TTA TAG C-3´; CpRap2.1 (forward) 5´-AAA AAG CAG GCT TCA CCA TGG AAG GAG AGT GTT CG-3´ and (reverse) 5´-AGA AAG CTG GGT CTT CAT CGG AAG TTT CCG G-3´; and CpRap2.10 (forward) 5´-AAA AAG CAG GCT TCA CCA TGG AGG GTG GAG CGG AG-3´ and (reverse) 5´-AGA AAG CTG GGT GCT CGC CAT CCG AAC TAT CTG GGT C-3´. Gateway BP Clonase® (Invitrogen, http://www.invitrogen.com) was used to recombine the *CpRap2*.*4a*, *CpRap2*.*4b*, *CpRap2*.*1*, and *CpRap2*.*10* genes with the flanking attB sites into the attP sites of the pDONR™221 vector (Invitrogen, http://www.invitrogen.com). Subsequently, the four CpRAP genes were transferred into the binary vector PK7FWG2.0 [38]; (http://gateway.psb.ugent.be/information), which contains the attR sites, using the Gateway LR Clonase® (Invitrogen, http://www.invitrogen.com). The binary vector PK7FWG2.0 contains the kanamycin resistance gene (*nptII*) under the control of the 35S promoter of cauliflower mosaic virus and the reporter gene for GFP.

### Overexpression of papaya Rap2 genes in tobacco plants

The constructed binary vectors that contained specific RAP2 genes (*CpRap2*.*4a*::gfp, *CpRap2*.*4b*::gfp, *CpRap2*.*1*::gfp, *CpRap2*.*10*::gfp) were introduced into *Agrobacterium tumefaciens* EHA 105 cells. *N*. *tabacum* (*Nicotiana tabacum* L. cv. SR1) plants were transformed according to the method described previously [39]. A disk of leaves of *N*. *tabacum* was prepared using a sterile cork border (8 mm) and placed onto preculture medium (MS salts, 3% sucrose, 10-ml/l Vitamins B5, 1-mg/l Benzilaminopurine (BAP) and 3-g/l Gelzan. These explants were incubated at 25°C under a photoperiod of 16 h light/8 h dark for 24 h. The explants were transformed using the vacuum infiltration method and cocultived at 25°C in the dark for 3d and then transferred to selection medium (MS salts, 3% sucrose, 10-ml/l Vitamins B5, 1-mg/l Benzilaminopurine (BAP), 0.1-mg/l NAA, 3-g/l Gelzan, 200-mg/l Timetin and 150-mg/l kanamycin).

### PCR analysis of putative transgenic plants

Genomic DNA of putative transformed plants (100 ng) was used for a PCR reaction to detect the presence of the *nptII* transgene using the primer pairs, *nptII*-forward (5´-ATG ATT GAA CAA GAT GGA TTG C-3´) and *nptII*-reverse (5´-TCA GAA CTC GTC AAG G-3). Specific RAP2 gene primers were tested on each transgenic plant to improve the quality of the analysis. PCR products were run on a 2% agarose gel with ethidium bromide and visualized on a Molecular Imager® Gel Doc™ XR+ System.

### Stress tolerance tests of tobacco transgenic plants

Transgenic seeds from twelve transgenic lines *N*. *tabacum* were grown in selection medium (MS salts, 3% sucrose, 10-ml/l Vitamins B5, 200-mg/l Timentin, 150-mg/l kanamycin, and 3-g/l Gelzan) according to a previous study [39]. Transformed and non-transformed seedlings were transferred in a potting mixture of Horti Pearl, Sunshine, Vermiculite, and Peat moss (2:2:2:1) and then grown under a 16 h light/8 h dark photoperiod regime at 25°C for six weeks. Up to 45 days after germination (DAG) the plants were used for a temperature tolerance test. For cold stress treatment, the 45 DAG plants were transferred to the same potting mixture and incubated for 30 days at 4°C. In the case of high temperature stress treatment, 45 DAG plants were subjected to 40°C for 12 days. The control group was kept at room temperature (25°C).

### Cellular localization of the green fluorescent signals in transgenic plants

GFP fluorescence (excitation filter 488 nm, emission filter band pass of 505–530 nm) was analyzed using a confocal laser-scanning FV100 Olympus microscope. DAPI staining of *N*. *tabacum* transgenic plants was performed in order to determine the location of the nuclei in the cells. Non-transformed plants were used as a GFP control of protein localization.

### Histology and immunofluorescence microscopy

The plant tissue was fixed in tubes containing FAA with aspiration for 24 h. They were dehydrated through an ethyl alcohol series and embedded in paraffin (melting point 54–56°C) with a graded series of tertiary butyl alcohol. The paraffin blocks were sectioned serially at 5 μm thickness using a microtome. The deparaffinization was carried out with four washes with Histology grade Xylene for 2 min and by removal of xylene with absolute ethanol. Seventy percent ethanol followed by water for 1 min each. Tissue was permeabilized with 0.1% Triton X-100 in PBS for 15 min, respectively. After washes with PBST they were either incubated with anti-GFP from Anti-GFP antibody (ab6556) from Abcam. Secondary antibodies goat anti-rabbit IgG conjugated with Alexa 647 from Invitrogen (21245) using an excitation wavelength of 620 nm or 660 nm. After being washed for 30 min with PBST cells were mounted with moviol (DAPI-DABCO). Images were acquired using a confocal microscope FV100 Olympus with 60X (NA 1.4) oil immersion objective lens. In each experiment, the images of all samples were acquired with the same parameters of acquisition to ensure their comparability. To assess the distribution of all transgenic plants that expressed GFP fused proteins in the tissues. The GFP fused proteins were analyzed in a 500–530 nm emission filter in the image was color-coded in green. DAPI emission 425–475nm channels were color-coded in blue. For comparison all images were acquired with the same excitation and detection parameters without reaching signal saturation.

### *In vitro* translation in the sap of *Carica papaya* L. cv. Maradol

We used GST-tagged PLCδ1PHD mRNA to test in vitro translation capability of the sap. Sap was extracted by cutting 1 to 2 mm of the apical bud, from *C*. *papaya* plants of six weeks old, suspending at storage buffer (24-mM HEPES buffer pH 7.4, 101-mM potassium acetate, 4.2-mM magnesium acetate, 11-mM DTT and 1-mM spermidine). The RNA was synthesized in vitro from GST-tagged PLCδ1PH (1–140; pGST3) a vector provided by Dr. Hitoshi Yagisawa [40]. 25 units of T7 RNA polymerase was incubated at 37°C for 1 hour in buffer containing 50 mM Tris-HCl pH 7.5, 15 mM MgCl, 2,5 mM dithiothreitol (DTT), 2 mM spermidine and 2mM dNTPs with 10 pmoles of vector. After incubation the RNA was purified by Qiagen RNeasy mini kit and verified by agarose gel electrophoresis. The translation reactions were performed with a 0.08 mM amino acid mixture, 75 mM potassium acetate, 1 μl of RNasin (40 u/μl), 0.5 μg/μl of GST-PLCδ1PHD RNA and 25 μl of *C*. *papaya* sap in a total volume of 50 μl with nuclease-free water. The reaction was incubated at 30°C for 2 hours. For Immunoblot analysis, the reaction was stopped with laemmli buffer and heated at 100°C for two minutes to denature the proteins and then loaded in a 10% SDS-PAGE gel. Bacterially expressed GST-PLCδ1PHD was used as a positive control. The SDS-PAGE was blotted into nitrocellulose paper block with 5% milk in PBST for one hour and incubated for 2 hours with anti-GST antibody (Abcam ab19256).three subsequent washes with PBST were carried out 10 min each and incubated for 2 hours with IRDye goat anti-rabbit antibodies and analyzed by Odyssey Infrared Imager 9120 (LI-COR Biosciences).

### Nicotiana tabacum L. Grafting

Three independent lines Rap2.4a transgenic plants from the twelve lines of *N*. *tabacum* L. were grown in a greenhouse until the F1 generation was obtained. F1 seeds were germinated for 60 days. The grafts between the non-transformed and transformed plants were performed using the transgenic tobacco plants as rootstock and the wild-type tobacco plants as scion. For the grafting, straight cuts were made into the rootstock, and then the apical section of the tobacco wild-type plant was inserted until it fit snugly. The cut was wrapped with polythene strips for one week.

### Photosynthesis measurements in transgenic plants

The photosynthetic net rate (*P*n) and stomatal conductance (*G*s) were measured using a portable infrared gas analyzer (Li-6400XT, Licor, Lincoln, NE, USA). High temperature (40°C) was measured at 0, 4, and 7 days. The analyzer was equipped with a clamp-on leaf cuvette that exposed 6 cm^2^ of leaf area. Light, temperature, and humidity were 180 μmol m^-2^ s^-1^ (Li-6400-02B LED), 25± 1°C and 70%, respectively and a CO_2_ concentration of 400 μmol mol^-1^. The readings were determined within 60 seconds of having locked up the leaf chamber. Measurements were made on four randomly selected plants of each treatment.

### Scanning electron microscopy

To study the promotion of stomatal closure by ABA, *Nicotiana tabacum* seeds were sown on ABA-free medium for 4 d after stratification, and then the tobacco *in vitro* plantlets were transferred into a hydroponic liquid MS medium (Murashige and Skoog, 1962), and after 4 d of acclimation, 50 μM of ABA was added to the hydroponic medium for 2 days of treatment. Stomatal aperture was observed from the central regions of leaves from the abaxial epidermis using Scanning Electron Microscopy (SEM). This experiment was carried out as follows: 1 cm of sample was fixed in 2.5% glutaraldehyde for 48 h, rinsed several times in buffer solution (0.2 M sodium phosphate, pH 7.2), and dehydrated in serial ethanol concentrations (v/v) of 30%, 50%, 70%, 85% and 96%. All these dehydrated rinses were sequentially followed by a wash in absolute ethanol for 60 min. The critical drying point was performed at 1072 psi/31°C (Samdri®-795 tousimis). The samples were then mounted on metallic stubs with carbon conductive adhesive tape (Electron Microscopy Science) and sputter coated with a 150 Å gold layer (Denton Vaccum Desk II). Stomatal aperture percentage was calculated for the abaxial epidermis at a magnification of 200×(0.1213 mm^2^). Length of the guard cells was also measured in five stomata per field. Counts and measurements were done in ten fields of each leaf. Sample analysis and image recording were made using a Scanning Electron Microscope (Jeol, JSM-6360LV).

### Statistical analyses

For the photosynthesis measurements each bar represents the average measurement from three independent samples for each condition. Wild type-wild type and *CpRap2*.*4a* transgenic gene-wild type tobacco plants were taken and indicated by different letters depending on their differences. The data was analyzed by one-way analysis of variance (ANOVA) followed by the comparison of mean values using the Tukey's test at P<0.01 in Statgraphics Centurion (StatPoint Technologies, Inc., Warrenton, VA). Data were presented as mean ±SD error of three determinations per sample.

## Results

### Genome-wide identification of AP2/ERF sequences and phylogenetic relationships

The retrieval of 832 AP2/ERF sequences from eight full-genomes (see Materials and Methods) by means of HMM was used to identify AP2/ERF homologs in papaya tree [41]. We detected 78 papaya AP2/ERF sequences, and we used the prefix CpERF to name the papaya AP2/ERF sequences. Two full-length cDNA sequences in papaya are closely related to AtRAP2.1 and AtRAP2.10, respectively, which are not described in the proteome file from the Phytozome database. These predicted sequences were obtained using the FGENESH+ suite [42] with a predicted coding sequence ranging from 157 to 181 amino acids, and they were named CpERF79 and CpERF80, respectively. These two new sequences bring the number of CpERF sequences to 80. No discrepancies were found between the PCR amplicons of these two genes and the FGENESH+-predicted coding sequences [42]. The complete list of the AP2/ERF TF family from papaya tree is available in S1 Table.

**Fig 1:**
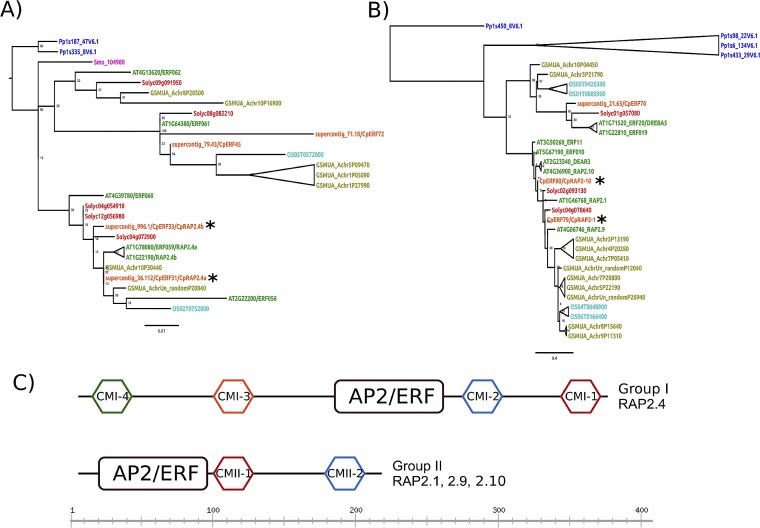
Maximum likelihood phylogenetic trees of the RAP2.4 and RAP2.1/2.9/2.10 subgroup proteins of the AP2/ERF transcription factor family in eight plant species. A) Phylogenetic reconstruction of the RAP2.4 subgroup of the group I AP2/ERF TFs. B) Phylogenetic reconstruction of the RAP2.1/2.9/2.10 subgroup of group II AP2/ERF TFs. Species names are color coded as follows: blue–Moss, pink–pseudofern, green–Arabidopsis, orange–papaya, red–tomato, yellow–banana, and cyan–rice. C) Schematic representation of the identified conserved motifs in each analyzed subgroup. Sequences of *P*. *patens* were used as the out-group. Black asterisks indicate the papaya AP2/ERF proteins analyzed in this work.

Phylogenetic trees of the retrieved AP2/ERF sequences were constructed from the AP2 domain, and they were grouped according to a previous study [6]. Four CpERF sequences were clustered in the group I, subgroup of RAP2.4 genes (CpERF31, CpERF33, CpERF45, and CpERF72), and three CpERF sequences were clustered in the group II, subgroup of RAP2.1/2.9/2.10 (CpERF70, CpERF79, and CpERF80; Fig 1A and 1B). We performed an amino acid analysis based on a sequence alignment for these two subgroups, RAP2.4 and RAP2.1/2.9/2.10, to determine the conserved motifs along the plant lineages (see Fig 1C). The four CMI motifs reported by Nakano e*t al*. [6] were conserved in a RAP2.4 subgroup (S1 Fig), and two CMII-reported motifs were found in the RAP2.1/2.9/2.10 subgroup (S2 Fig).

Orthologous proteins of interest were calculated by means of reciprocal best Blast hits (BBH), [43] using the entire set of papaya AP2/ERF sequences against the Arabidopsis AP2/ERF retrieved sequences. For the RAP2.4 subgroup, CpERF31 protein is orthologous to AtRAP2.4 (AT1G78080), with three possible paralogous sequences (AT1G36060/ERF055, AT1G22190/RAP2-4b, and AT5G65130/ERF057), and CpERF33 protein is orthologous to ERF60 (AT4G39780), with two possible paralogous sequences (AT2G22200/ERF056 and AT4G13620/ERF062). For the RAP2.1/2.9/2.10 subgroup, CpERF80 protein is orthologous to AtERF10 (AT5G67190), with three possible paralogous sequences (AT4G36900/RAP2-10, AT3G50260/ERF011, and AT2G23340/DEAR3), and CpERF79 protein did not show reciprocal BBH, but was the closest related to the AtRAP2.1 protein.

CpERF31 and CpERF33 proteins were called CpRAP2.4a and CpRAP2.4b, respectively, due to their proximity to AtRAP2.4 (group I; see Fig 1). CpERF79 and CpERF80 were called CpRAP2.1 and CpRAP2.10, respectively, because of their similarity to the Arabidopsis proteins AtRAP2.1 and AtRAP2.10, from the AP2/ERF group II.

### Expression analysis of *CpRap2* genes in different plant tissue and sap under abiotic stress conditions

**Fig 2:**
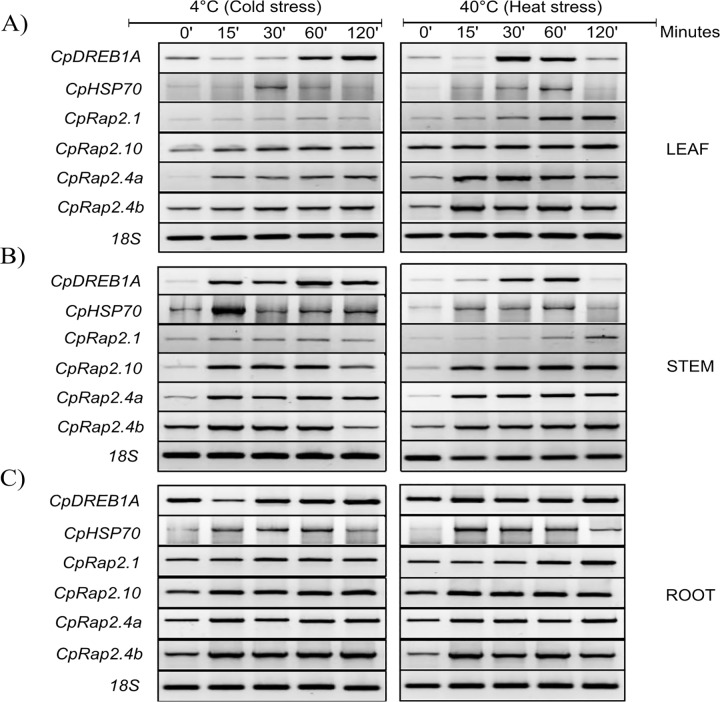
Semi-quantitative reverse transcription PCR (RT-PCR) analysis of the different CpRap genes exposed at extreme temperature stress conditions from *Carica papaya* tree. A) RT-PCR analysis of CpRap genes exposed to 4°C and 40°C during different times of treatment in leaves of *C*. *papaya*. B) RT-PCR analysis of CpRap genes exposed to 4°C and 40°C during different times of treatment in stems of *C*. *papaya*. C) RT-PCR analysis of CpRap genes exposed at 4°C and 40°C during different times of treatment in roots of *C*. *papaya*. The quantification values of CpRap genes were normalized with that of the 18S gene. All analyses were performed with three biological replicates.

Molecular analysis of the expression of *CpRap2* genes was performed to detect the expression of *CpRap2*.*4a*, *CpRap2*.*4b*, *CpRap2*.*1* and *CpRap2*.*10* genes, and the 18S gene was used as a reference. Fig 2 shows the RT-PCR levels of the *CpRap2*.*4a*, *CpRap2*.*4b* and *CpRap2*.*10* genes obtained in the leaf and stem during different stress treatments of heat (40°C) and cold (4°C) (Fig 2A and 2B). The transcript level of these genes (*CpRap2*.*4a*, *CpRap2*.*4b*) were induced in the leaf and stem by heat (40°C) and cold (4°C) stress (Fig 2A, 2B and 2C) and S2 Table.

Previous studies found that the *AtDreb1A* (dehydration-responsive element-binding protein 1A) gene is expressed in response to drought and low temperature, and the *AtHsp70* (70-kilodalton heat shock proteins) gene is expressed in response to high temperature. Therefore, we isolated the orthologous genes from *C*. *papaya* var. Maradol and used them as positive controls for the extreme temperature experiments. The *CpDreb1A* gene in the leaf and stem is differentially expressed in response to extreme temperatures (Fig 2A and 2B). *CpDreb1A*-type gene transcript accumulated as exposure time increases to stress unlike the behavior of this transcript expression in roots (Fig 2C). In comparison the transcript expression profile of *CpHsp70*-type gene clearly shows a higher yield in roots than in other tissues of the papaya plants (Fig 2C). The expression of both *CpHsp70* and *CpDreb1A*-type genes decreases when the plants have reached at 120 minutes of exposure to stress. This decrease in expression occurs when seedlings were greatly affected already by stress. It is important to note that this does not happen with the *CpDreb1A*-type transcription in root. The transcript accumulation of the *CpDreb1A*-type gene was diminished in leaf and stem, but not in root of the papaya seedlings plant. The expression of the *CpHsp70* gene was also differential accumulated in the root of the papaya seedlings (Fig 2A, 2B and 2C).

**Fig 3:**
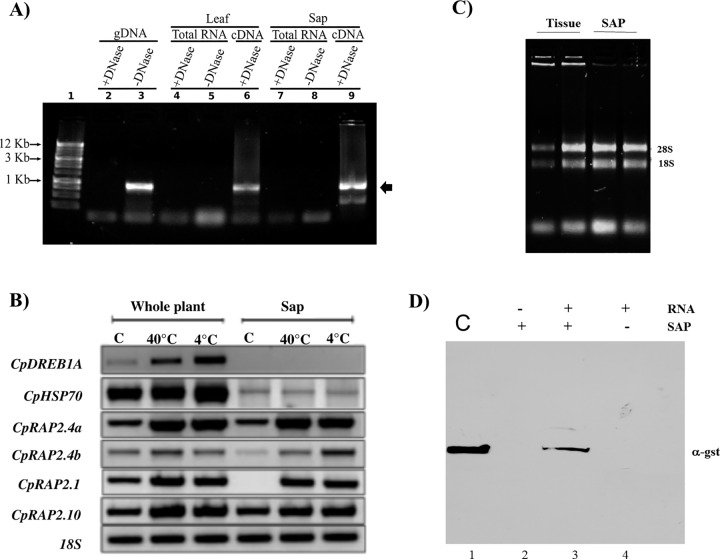
Transcriptional expression of different genes in the sap of *Carica papaya* and *in vitro* translation from sap. A) PCR from Genomic DNA, Total RNA, and cDNA of leaf and sap in the absence or presence of DNase I. Nomenclature: 1. 1 Kb ladder (Promega); 2. PCR of DNase I treated Genomic DNA; 3. PCR of Genomic DNA; 4. PCR of DNase I treated total RNA; 5. PCR of total RNA; 6. PCR of cDNA synthesized from DNase I treated total RNA; 7. PCR of DNase I treated Sap RNA; 8. PCR of Sap RNA; 9. PCR of cDNA synthesized from DNase I treated RNA sap. B) RT-PCR analysis of CpRap genes in sap or the whole plant of *C*. *papaya* exposed to 4°C and 40°C. Quantification values of CpRap genes were normalized with the 18S gene. All analyses were performed with three biological replicates. C) Total RNA, comparison of tissue vs sap separated on a 1% agarose gel after electrophoresis with ethidium bromide. 28 and 18 S rRNA are mark by the arrows. D) Immunoblot analysis after *in vitro* translation of GST-PLCd1PHD with sap of *C*. *papaya*. Recombinant GST-PLCd1PHD was loaded as a control (C), 20 μl of the in vitro translation reactions were loaded, minus RNA added to the sap, with RNA and sap and RNA only.

We carried out PCR and RT-PCR and verify that no DNA contamination responsible for the amplifications from our Total RNA or RNA form sap extractions (Fig 3A). We detected the RNA of *CpRap2*.*4a*, *CpRap2*.*4b*, *CpRap2*.*1* and *CpRap2*.*10*-type gene in the sap as well as in the complete tissue of the plant. *CpRap2*.*4a*, *CpRap2*.*4b* and *CpRap2*.*1* gene expression increased in both sap and tissues, while other genes, such as *CpDreb1A* and *CpHsp70*, were only found in the tissue, also suggesting a lack of contamination of RNA from the tissue into the sap (Fig 3B and S3 Table). Transcript accumulation in control seedlings have a basal expression, however in response to stress at 4°C or at 40°C, there is a transcript accumulation, suggesting that CpRAP2.1, CpRAP2.4a and CpRAP2.4b are involved in setting the signals for plant survival at extreme temperatures. The gene specificity of RT-PCR was confirmed by sequencing all of the RT-PCR products. The results for the *CpRap2*.*1* gene were different in sap as compared to the whole plant indicating a selective mechanism between the RNA that is send to the sap. Furthermore upon checking the RNA extracted from sap we found ribosomal RNA in the extracts from sap as seen in Fig 3C. Therefore we tested the idea that protein translation could occur in the sap and carried out in vitro translation reactions using sap to create a foreign protein GST PLCd1PHD (Fig 3D). Surprisingly we were able to translate a protein using sap of *C*. *papaya* from an in vitro synthetized RNA that codifies for GST PLCd1PH D, Other results from the in vitro translation reactions from sap included translation and purification of 6his-fibrilarin a nucleolar protein S3 Fig and S1 File. Furthermore, added mRNA of *gfp-Rap2*.*4a* to wound embryonic also showed some cells translating the foreign mRNA and incorporating it in the cells S4 Fig and S1 File.

### Stress tolerance assay of overexpressing CpRAP2 proteins in tobacco plants

**Fig 4:**
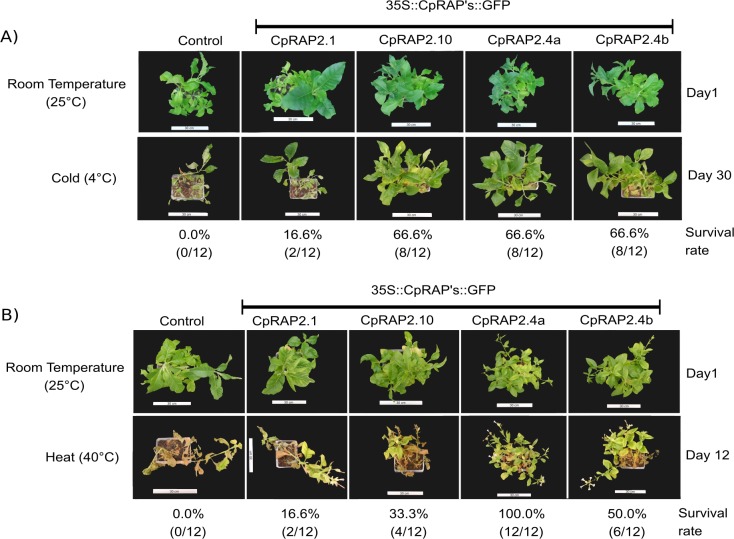
Extreme temperature tolerance of tobacco transgenic plants that carry in their genome different CpRap genes controlled by the 35S promoter. A) Phenotypes are shown for 12 transgenic tobacco plants with the *CpRap2*.*1*, *CpRap2*.*10*, *CpRap2*.*4a*, and *CpRap2*.*4b* gene under low temperature stress (4°C) or at room temperature (25°C). B) Phenotypes are shown for 12 transgenic tobacco plants with the *CpRap2*.*1*, *CpRap2*.*10*, *CpRap2*.*4a* and *CpRap2*.*4b* gene under high temperature stress (40°C) or at room temperature (25°C). Wild-type tobacco plants were used as a control.

The phenotype analysis of F1 transgenic and non-transformed tobacco plants under the stress of extreme temperatures (4°C or 40°C) is shown in Fig 4, where a single plant is a typical representative from each population. We used 12 plants for each experiment from 3 selected lines for each transgene. All plants were exposed at 4°C for 30-day. Fig 4A shows that non-transformed plants did not survived, while transgenic tobacco plants overexpressing the *CpRap2*.*1* gene had a survival rate of 16.6%, and the *CpRap2*.*10*, *CpRap2*.*4a* and *CpRap2*.*4b* genes showed survival rates of 66.6% (Fig 4A). High temperature stress treatments (40°C exposure for 12 days) resulted in the lack of survival of the non-transformed plants, while transgenic tobacco plants carrying the *CpRap2*.*1*, *CpRap2*.*10*, *CpRap2*.*4a* and *CpRap2*.*4b* genes had a survival rate of 16.6, 33.3, 100 and 50%, respectively (Fig 4B). Tobacco plants that overexpressed CpRAP2.4a or CpRAP2.4b showed the particular phenotype of all of the adult leaves being slightly marked by a green and chlorotic (yellow green) color after the treatment at 4°C or 40°C (Fig 4A and 4B); they also began to flower after 12 days of high temperature treatment. Tobacco plants that overexpressed CpRAP2.4a or CpRAP2.4b showed a normal plant growth, however they presented some wilting or chlorosis. While under treatment their stems did not showed a signs of death tissue and maintained their vigor after 30 days treatment at 4°C. When the plants were incubated at 40°C for 12 days the tobacco plants that overexpressed CpRAP2.4a or CpRAP2.4b showed a reduced plant growth, with an abnormal curling and crinkling of some leaves, including the reduction expansion of leaves, wilting, chlorosis and their stems showed some loss of vigor.

Furthermore we compared seed germination of the wild-type (Wt), CpRAP2.4A and CpRAP2.4B overexpression transgenic lines in the presence and absence of Abscisic acid (ABA). ABA is a phytohormone critical for plant growth and development and plays an important role in integrating various stress signals and controlling downstream stress responses. The results are shown in S5 Fig, where the expression of both genes in tobacco allows their germination under ABA presence.

### Localization of the CpRAP2 proteins in transgenic tobacco seedlings

**Fig 5:**
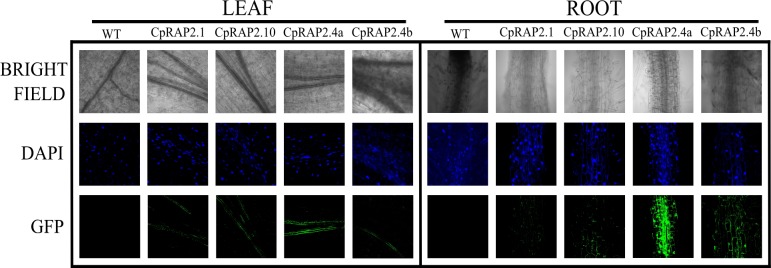
Subcellular accumulation patterns of CpRAP proteins fused to GFP in *Nicotiana tabacum* plants. The leaves or roots of tobacco seedlings after 8 days of germination were taken under different light sources Transmitted light is observed in the horizontal first line, DAPI staining is observed in the second horizontal line and GFP is observed in the third horizontal line. DAPI staining or GFP fluorescence of leaf or root is shown for tobacco transformed plants that they carry different CpRAP genes (the *CpRap2*.*1*, *CpRap2*.*10*, *CpRap2*.*4a* and *CpRap2*.*4b* gene) in their genome. The fluorescence of GFP and DAPI staining were taken at 40X on an Olympus FV1000 confocal microscope. Wild-type tobacco plants were used as a control.

In order to identify the sub-cellular localization of the CpRAP2.1, CpRAP2.10, CpRAP2.4a and CpRAP2.4b proteins, they were fused with a green fluorescent protein (GFP). Each construct was expressed constitutively under the control of a cauliflower mosaic virus (CaMV) 35S promoter. Transgenic tobacco seedlings overexpressing the chimeric CpRAP2.1::GFP, CpRAP2.10::GFP, CpRAP2.4a::GFP or CpRAP2.4::GFP proteins were analyzed for GFP fluorescence with a confocal microscope (Fig 5, larger files can be observed in S6 Fig). The nuclear location of CpRAP2.1, CpRAP2.10, CpRAP2.4a, and CpRAP2.4b protein was confirmed by GFP and DAPI merged images showing a complete match (Fig 5). GFP was observed in the leaves of transgenic tobacco with CpRAP2.1, CpRAP2.10, CpRAP2.4a or CpRAP2.4b in the sieve elements (SE), which are part of the phloem.

Micrographs taken from transmitted light (bright-field microscopy) in the leaves and roots of different lines of tobacco seedlings (control, CpRAP2.1, CpRAP2.10, CpRAP2.4a and CpRAP2.4b) localized to the veins of the leaves. Only the transgenic tobacco expressing CpRAP2.4a::GFP shows a strong GFP signal in phloem tissue where it can distinguish the sieve elements. Micrographs of the root apices show a different level of accumulation of green fluorescent protein in transgenic tobacco. The accumulation of GFP in CpRAP2.4a was very strong, and the accumulation of GFP protein was observed in the nuclei and the cell membranes of the roots. For transgenic tobacco seedlings with CpRAP2.1 or CpRAP2.10, the accumulation of GFP protein was restricted to around the cell membrane and was not present in the nuclei of the cells. These results and the *in silico* analysis suggests to us that CpRAP2.4a is a nuclear protein that possibly serves as a transcription factor.

### Long-distance transport assay of *CpRap2*.*4* mRNA by tobacco grafting

**Fig 6:**
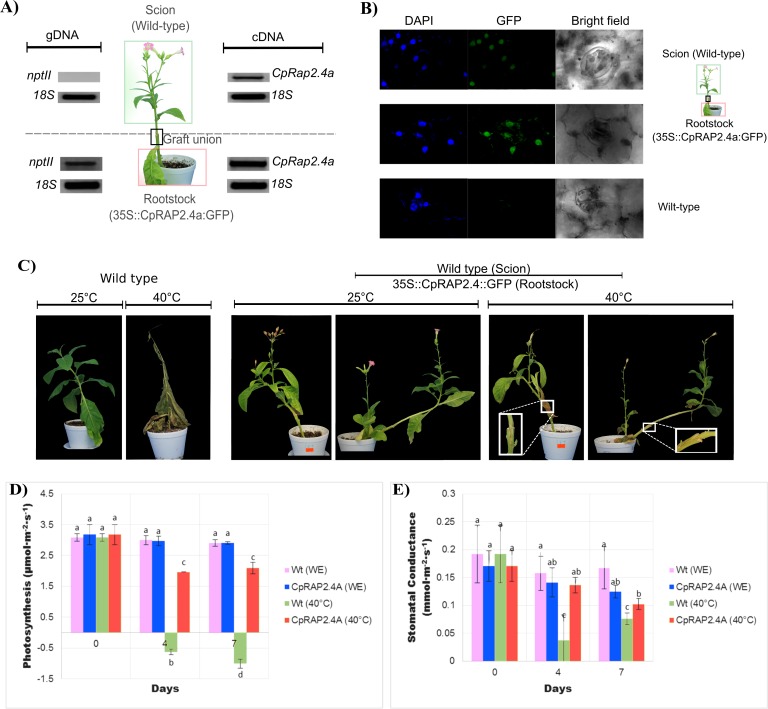
Long-distance transport assays of CpRap2.4a by tobacco grafting. A) Genomic DNA (left section) was isolated from wild-type (upper section; Scion) or transgenic plants (below section; Rootstock) for amplification using PCR. The nptII primers were used to identify the neomycin phosphotransferase II (*nptII*) that is the selection marker of plants contained in the T-DNA. Total RNA (right section) was isolated from the wild-type tobacco (upper section; Scion) or from the transgenic tobacco plant (below section; Rootstock) for amplification using RT-PCR. CpRap2.4a primers were used to identify the accumulation of the *CpRap2*.*4* transcript. Quantification values of *nptII* or the *CpRap2*.*4a* gene were normalized with the 18S gene. B) Subcellular accumulation patterns of CpRAP2.4a proteins fused to GFP in a tobacco plant graft. Leaf sections of a wild-type tobacco (horizontal first line) were taken under different light sources (DAPI staining is observed in the first vertical line, GFP is observed in the second vertical line and transmitted light is observed in the third vertical line). DAPI staining or GFP fluorescence in the leaf is shown for transformed tobacco plants (second horizontal line) that carry the *CpRap2*.*4a* gene in their genome. The fluorescence of the GFP and DAPI staining was taken at 40X by an Olympus FV1000 confocal microscope. Wild-type tobacco plants were used as the control (third horizontal line). C) Phenotypes of the tobacco plant grafts with the *CpRap2*.*4a* gene under high temperature stress (40°C) or at room temperature (25°C). Wild-type tobacco plants were used as a control. D) and E) Physiological effects of *CpRap2*.*4a* rootstock overexpressing tobacco plants under high temperature treatments in the photosynthetic activity and stomatal conductance from leaves of the wild type tobacco plants. *N*. *tabacum* transgenic (Rootstock) and wild type tobacco (Scion) plants were treated at 40°C during different times. Data was analyzed by one-way analysis of variance (ANOVA) followed by the comparison of mean values using the Tukey's test at P<0.01. Data were presented as mean ±SD error of at least three independent determinations per sample. Different letters indicate significant differences. Units: [photosynthesis = μmol.CO2.m^-2^.s^-1^; Stomatal conductance = mmol.m^-2^.s^-1^].

Movement of CpRAP2.4a::GFP was investigated by grafting experiments with tobacco transformed plants with the constructs CpRAP2.4a::GFP as a rootstock and non-transformed tobacco plants as scion (Fig 6A). Genomic DNA from tobacco transformed and wild-type plants were used for PCR for CpRAP2.4a gene. Wild-type tobacco plant did not contain the amplicon for the neomycin phosphotransferase II (*nptII*) gene, which is contained in the T-DNA that was inherent to the transgenic explants (Fig 6A). Total RNA from the leaves of each graft was obtained and RT-PCR was performed using the specific primer for the CpRAP2.4a gene. We observed that the tobacco transgenic plant with the construct CpRAP2.4a::GFP in the rootstock transferred the CpRAP2.4a::GFP RNA through the vascular conduits of the plant to the wild-type scion.

The CpRAP2.4a protein was also localized in the nucleus of the wild-type scion (Fig 6B). DAPI was used to verify that it was in the nucleus, and both the transformed tobacco leaf with the *CpRap2*.*4a* and the wild-type leaf from the scion tobacco leaf showed nuclear localization. Although the wild-type scion signals were clearly lower. Control wild-type leaves did not show GFP fluorescence (Fig 6B). Graft tobacco plants were exposed to heat-induced stress (40°C), where non-transformed tobacco was affixed to the transformed plants (rootstock, Fig 6C); we observed that the rootstocks confer a capacity of the grafts to tolerate heat stress in conditions that wild-type tobacco plants did not tolerate (Fig 6C). Also the phenotype of the grafted plants included the presence of yellowish-green leaves, and their stems were evenly turgid despite the stress treatment. Control non-transformed tobacco plants show the leaves and stems were necrotic with a brown color, with a high loss of turgor during the stress treatment. Their leaves showed an abnormal curling and crinkling, which may lead to necrosis and their stems showed a progressive death, with loss of vigor. Wild type and CpRAP2.4 transgenic plants showed the same photosynthesis and stomatal conductance when no stress was added to the plants. After 4 days wild type plants had a negative photosynthetic rate and a very low stomatal conductance while the wild-type scion with the CpRAP2.4a showed a small reduction in photosynthetic rate and no significant difference in stomatal conductance (Fig 6D). This trend was maintained up to the 7 days of the experiment indicating the CpRAP2.4a rootstock those provide the wild type scion a change in physiological response to high temperature (40°C).

**Fig 7:**
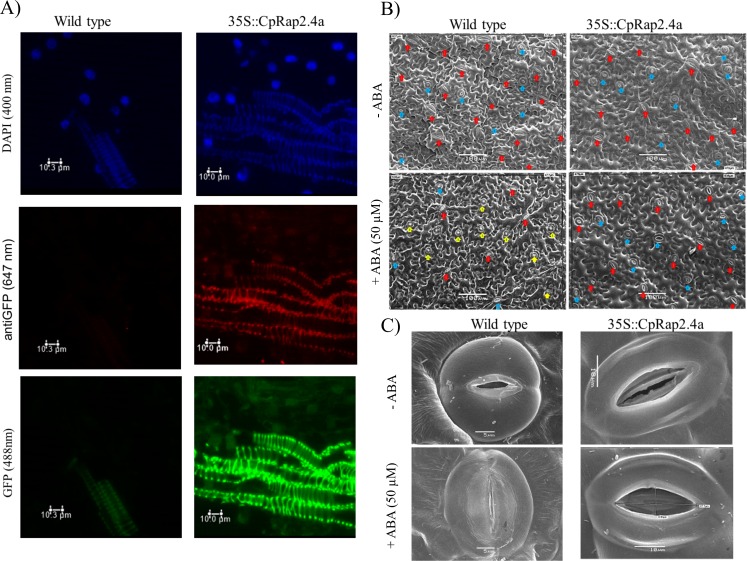
RAP2.4a localization in leaf cells and its effect on stomatal aperture. A) Immunocolocalization studies show the localization of GFP-35S::CpRAP2.4a in transgenic plants in nucleus and sieve elements. Lignin from sieve elements shows autofluorescence at 480nm but not at 620 nm. B) SEM of stomatal aperture in wild type tobacco plants versus transformed tobacco plants (35S::*CpRap2*.*4a*). Arrows show stomata in blue (open) and red (close) for wild type and GFP-35S::CpRAP2.4a in transgenic plants in presence or absence of ABA. Yellow arrows indicate rounded closed stomata only seen in wild type plants in the presence of 50 μM ABA. C) Phenotype of Stomata of leaves from the abaxial epidermis of the wild type tobacco plants (Wt) versus transformed tobacco plants (35S::*CpRap2*.*4a*) in presence or absence of ABA.

The stomata of wild-type plants had smaller guard cells and the aperture percentage were approximately 66% lower than those in *35S*::*CpRap2*.*4a* plants in ABA treatment (Fig 7). Furthermore, the size of stomata were 36% larger in *35S*::*CpRap2*.*4a* plants. Wild type plant under the presence of ABA showed to be very tightly closed in a round configuration marked with yellow arrows which was not seen in the in *35S*::*CpRap2*.*4a* plants under the same treatment. Therefore, CpRAP2.4a functions as a negative regulator of ABA-mediated stomatal closure. Different results were obtained by Lin, Park [12] where the RAP2.4-ox plants and *rap2*.*4–1* mutant plants responded normally to ABA inhibition of seed germination and root elongation. These results suggest that enhanced extreme temperatures tolerance of the CpRAP2.4a plants is likely a combination of functions from the CpRAP2.4a as a transcription factor and having a role in maintain stomata open upon signaling like that of ABA pathway. Consistent with this notion, the expression of *CpRap2*.*4a* is up-regulated by extreme temperature treatment.

## Discussion

Plants have evolved a unique signaling pathway that takes advantage of connections in the vascular tissue as part of an elaborate long-distance communication system. The information sent through this pathway has been implicated in regulating development, responding to biotic stress, delivering nutrients, and as a vehicle commandeered by viruses for spreading infections [44]. Numerous full-length transcripts have been identified in the sieve elements of several plant species [17, 45–49]. Using a pyrosequencing approach, Bai and collaborators obtained 58,673 high quality ash sequences from pooled phloem samples of green, white, black, blue and Manchurian ash [21].

To understand this type of long distance signaling in plants, we studied genes from the AP2/ERF superfamily that encodes transcriptional regulators that serve a variety of functions in plant development and responses to biotic and abiotic stimuli [50]. Members of the AP2/ERF superfamily contain at least one AP2 domain, which consists of approximately 60 amino acids. Arabidopsis RAP2.4 has a single AP2 domain and was classified into the A-6 group [5] or into group I of the AP2/ERF TFs [6]. Our *in silico* analysis shows that the *CpRap2*.*4a* and *CpRap2*.*4b* genes of *C*. *papaya* contain this AP2 domain, and a sequence analysis grouped the *CpRap2*.*1* and *CpRap2*.*10* genes into group II of the AP2/ERF TFs. Some of these transcription factors have been characterized functionally with a phenotype that is tolerant of the presence of abiotic stress, similar to AtRAP2.4, AtRAP2.1 and AtRAP2.10 from *Arabidopsis thaliana* [12, 51–54]. *CpRap2*.*4a* and *CpRap2*.*4b* gene transcript accumulation remained constant in the leaves, stem and roots over a temporal course in response to stress caused by low or high temperatures, but not in plants without stress, similar to the result that was obtained in *Arabidopsis thaliana* with *Rap2*.*4B* and *Rap2*.*4* mRNAs after dehydration, exposure to low temperatures and osmotic stress. However, the expression of *Rap2*.*4B* but not *Rap2*.*4* was induced by heat stress [54, 55], which is in agreement with earlier findings from Lin, Park [12], where AtRAP2.4 overexpression enhanced tolerance to drought stress. In tobacco plants, the overexpression of the *CpRap2*.*4a* gene resulted in a survival rate of 66.6% during the stress caused by low temperatures and a rate of 100% for high temperatures. While the results obtained with the *CpRap2*.*4b* gene showed a lower survival (50%) during the stress caused by high temperatures.

Transcription factors are typically localized in cell nuclei where they bind DNA and activate transcription. However, CpRAP2.4a::GFP and CpRAP2.4a::GFP were expressed in transgenic tobacco in the vascular ducts of the ribbing of the leaves, and in young root tips. Interestingly the CpRAP2.4a or CpRAP2.4b protein was present in the sieve elements (SE), which are part of the phloem. We also observed them in the nucleus of leaf cells, similar to the results obtained by Lin, Park [12], where RAP2.4 from *A*. *thaliana* is located exclusively in the nucleus under both dark and white light conditions, suggesting that RAP2.4 is nuclear-localized because it contains a putative NLS with the amino acids 146–157 (KPTKLYRGVRQR) [54].

In the sap of *C*. *papaya* tree a differential transcript accumulation was observed for *CpRap2*.*4a* and *CpRap2*.*4b* genes in response to low and high temperatures. Given that ribosomal RNA was found in the phloem, we decided to perform an *in vitro* translation reaction. Rabbit reticulocyte lysate (RRL) or wheat germ lysate has been used successfully to translate protein in response to mRNAs from a variety of species, including mammals, plants and phage [56, 57]. Through *C*. *papaya*, we could determine that sap is able to translate the T7:GST- PlC PHd1D: mRNA, and this surprising result suggests that sap contains all of the proteins that are required in a translation system, which is similar to the wheat germ lysate that is commonly used in *in vitro* translation assay kits and may provide a mechanism for translating mRNA outside of the cells. Additionally, this mechanism may be hijacked by viruses for plant infection.

We detected the long distance transport of CpRAP2.4a protein and its mRNA from the rootstock of tobacco transgenic plant to the apex leaves of the non-transformed tobacco plant. Transport of the CpRAP2.4a protein to the tobacco transformed section, allows the plant to tolerate the stress caused by high temperatures, and CpRAP2.4a::GFP protein can be visualized in the tissue of tobacco non-transformed plants. Additionally, the movement of RAP2.4a::GFP protein and mRNA was also confirmed in injected *C*. *papaya* embryos. The recovery of photosynthetic net rate as well as stomata conductance under high temperature conditions, on tobacco scion from the transformed rootstock show a physiological alteration induced by stomatal regulation of gas exchange. Decreased photosynthetic rate is the result of stomatal and non-stomatal (biochemical) limitations [58, 59]. Plants react to water deficit with a rapid closure of stomata so it can avoid further loss of water during transpiration [60] resulting in restrictions on diffusion of CO_2_ in the leaf [60, 61]. Several studies have shown that decreased photosynthesis under drought stress can be attributed to perturbations of biochemical processes [61–63]. The reduction in *P*n due to high temperature could be attributed to both stomatal and non-stomatal limitations. Stomatal closure usually occurs before inhibition of photosynthesis and restricts CO_2_ availability at the assimilation sites in chloroplast. The effects of high temperature on photosynthesis are attributed directly to the stomatal limitations for gases diffusion, the recovery on the wild type scion from the transformed rootstock indicate an alteration either in the biochemical response or in the develop structure that has been developed from the overexpression of CpRAP2.4a transcription factor in the rootstock.

Previously, *StBEL5* RNA, a BEL1-like TF that is expressed in potato, was shown to be mobile (*Solanum tuberosum)* [64]. The BEL1-type TFs function in the floral pathway [65], inflorescence stem growth [66], stem cell fate [67], leaf architecture [68], ovule formation [69], and the establishment of egg cell fate in the mature embryo sac [70]. On the other hand, Lin, Sharma [71], demonstrated that the regulation of the *StBEL5* promoter in both stolons and roots is mediated by the phloem-associated movement of *StBEL5* mRNA from its transcriptional source in the leaves. This remarkable whole plant communication system involves light-induced transcription in the leaf, photoperiod-activated mobilization of the *StBEL5* mRNA through the phloem, and short-day regulation of *StBEL5* promoter activity in target organs growing underground in the dark. Recently Thieme and collaborators showed similar findings in *Arabidopsis*, with mRNA being transported to distant tissues. The study found that mobile transcripts are transported from the PED roots into flowering COL shoots after grafting. Additionally, the study showed how mobile transcripts from parasitic plants such as Cuscuta can get inside *A*. *thaliana* [72].

Here, long distance transport of CpRAP2.4a to leaves is observed. To date, several research groups have identified and characterized RAP2.4 proteins in various models, and the proteins have been described as regulatory elements in the response to different types of stress. However, until now, it was unknown whether any of the proteins in this family could be transported from one place to another within the plant via the vascular system. However, the route of internalization of these proteins into the cell remains to be determined, which is key to understanding how these proteins can be translocated into the cell. Furthermore, understanding the signals and properties of the mRNA that are sent into phloem would allow for the development of new technologies for the production of non-genetically modified plants to express certain characteristics of an internalized modified stock.

## Supporting Information

S1 Fig BOXSHADE analysis of the RAP2.4 subgroup belonging to group I of the AP2/ERF transcription factors. Thirteen sequences of different species were aligned to compare the conserved residues of the RAP2.4 clade. Colored boxes represent the AP2/ERF domain and conserved motif according to Nakano et al. (2006). (PDF)
(PDF)

S2 Fig BOXSHADE analysis of the RAP2.1/2.9/2.10 subgroup belonging to group II of the AP2/ERF transcription factors. Eleven sequences of different species were aligned to compare the conserved residues of the RAP2.1 clade. Colored boxes represent the AP2/ERF domain and conserved motif according to Nakano et al. (2006). (PDF)
(PDF)

S3 Fig For in vitro translation, we used RNA to test in vitro translation capability of the sap of the nucleolar protein fibrillarin. The RNA was synthesized in vitro from the pET15b::*fibrillarin* vector with T7 RNA polymerase at 37°C for 1 hour. The translation was performed with a 0.08-mM amino acid mixture minus methionine, 75-mM potassium acetate, 1 μl of RNasin (40 μ/ul), 2 μl of [35S] methionine at 10 mCi/ml, 0.5 μg/ul of *fibrillarin* RNA and 25 μl of *C*. *papaya* sap in a total volume of 50 μl with nuclease-free water. The sap was suspended in storage buffer (24-mM HEPES buffer pH 7.4, 101-mM potassium acetate, 4.2-mM magnesium acetate, 11-mM DTT and 1-mM spermidine). The reaction was incubated at 30°C for 2 hours. The sample was heated at 100°C for two minutes to denature the proteins and then loaded in a 10% SDS-PAGE gel. The gel was allowed to dry at 80°C for 90 minutes in a vacuum chamber and then was subjected to autoradiography. B) Purification of 6His-FIBRILLARIN after in vitro translation. Reaction was loaded on 50 ul Ni-Agarose resin washed and eluted with 250 mM Imidazole. Input (I), flow through (FT), washes (w1-w4) and elution’s (E1-E2) were loaded in the indicated lanes on a 12% PAGE, and silver stain for protein detection. (PDF)
(PDF)

S4 Fig Uptake and translation of *CpRap2*.*4a*::*gfp* in embryogenic callus. A) The embryogenic callus were wound 5 times with the tip of a insulin syringe that was used to add either the presence of 5 μg *CpRap2*.*4a*::gfp mRNA and then incubated for 3 days at 25°C in in MS medium containing glutamine at 0.4-mg/1 10-mg/1 2,4-D, 6% sucrose and 8-g/1 agar with the pH adjusted to 5.8. The embryos were mounted on slides with moviol with DAPI stain and analyzed the GFP fluorescence (excitation filter 488 nm, emission filter band pass of 505–530 nm). 2% of the cells showed nuclear GFP fluorescence from the embryonic tissue that was analyzed using a confocal laser-scanning microscope FV100 Olympus. DAPI staining was used to determine the location of the nuclei in cells. The experiment was carried out in triplicate (100 counted cells each time) and done on two independent times. B) Control callus without RNA incubation.
(PDF)

S5 Fig Seed germination rate of wild type, CpRAP2.4A and CpRAP2.4B tobacco overexpression lines. A) MS medium without ABA, B) MS medium supplemented with ABA 1 mM. (PDF)
(PDF)

S6 Fig Subcellular accumulation patterns of CpRAP proteins fused to GFP in *Nicotiana tabacum* plants. The leaves or roots of tobacco seedlings after 8 days of germination were taken under different light sources Transmitted light is observed in the horizontal first line, DAPI staining is observed in the second horizontal line and GFP is observed in the third horizontal line. DAPI staining or GFP fluorescence of leaf or root is shown for tobacco transformed plants that they carry different CpRap genes (the *CpRap2*.*1*, *CpRap2*.*10*, *CpRap2*.*4a* and *CpRap2*.*4b* gene) in their genome. The fluorescence of GFP and DAPI staining were taken at 40X on an Olympus FV1000 confocal microscope. Wild-type Tobacco plants were used as a control. A) leaf and B) root, tissues from CpRAP2.1 and CpRAP2.10 stained with DAPI. C) leaf and D) root, tissues from CpRAP2.4a and CpRAP2.4b. In S6D) merge from GFP and DAPI signals are merge to shows nuclear colocalization. (PDF)
(PDF)

S1 File Supplementary methods. Methods for RNA expression of CpRap2.4a::gfp, micropropagation of embryogenic culture of *Carica papaya*, and Uptake and translation of CpRap2.4a::gfp in embryogenic callus. (PDF)
(PDF)

S1 Table List of the AP2/ERF TF family in *Carica papaya*. We identified and retrieved 80 sequences with the AP2/ERF domain from *C*. *papaya* tree and that were named CpERFs. The groups were clustered according to Nakano *et al*. (2006). (PDF)
(PDF)

S2 Table Normalized densitometry from RT-PCRs from Fig 2. Each band was quantified and normalizes by dividing the value from the 0 time and then multiplying the value by 100. (PDF)
(PDF)

S3 Table Normalized densitometry from RT-PCRs from Fig 3E. Each band was quantified and normalizes by dividing the value from the 0 time and then multiplying the value by 100. (PDF)
(PDF)
