# Supplementary material for: RAP2.4a Is Transported through the Phloem to Regulate Cold and Heat Tolerance in Papaya Tree (Carica papaya cv. Maradol): Implications for Protection Against Abiotic Stress
Source: PLoS One. 2016 Oct 20;11(10):e0165030. doi: 10.1371/journal.pone.0165030 (PMC5072549; doi:10.1371/journal.pone.0165030)
Supplement: S2 Fig — Eleven sequences of different species were aligned to compare the conserved residues of the RAP2.1 clade. Colored boxes represent the AP2/ERF domain and conserved motif according to Nakano et al. (2006). (PDF) (PDF) [file pone.0165030.s002.pdf]

Figure S2

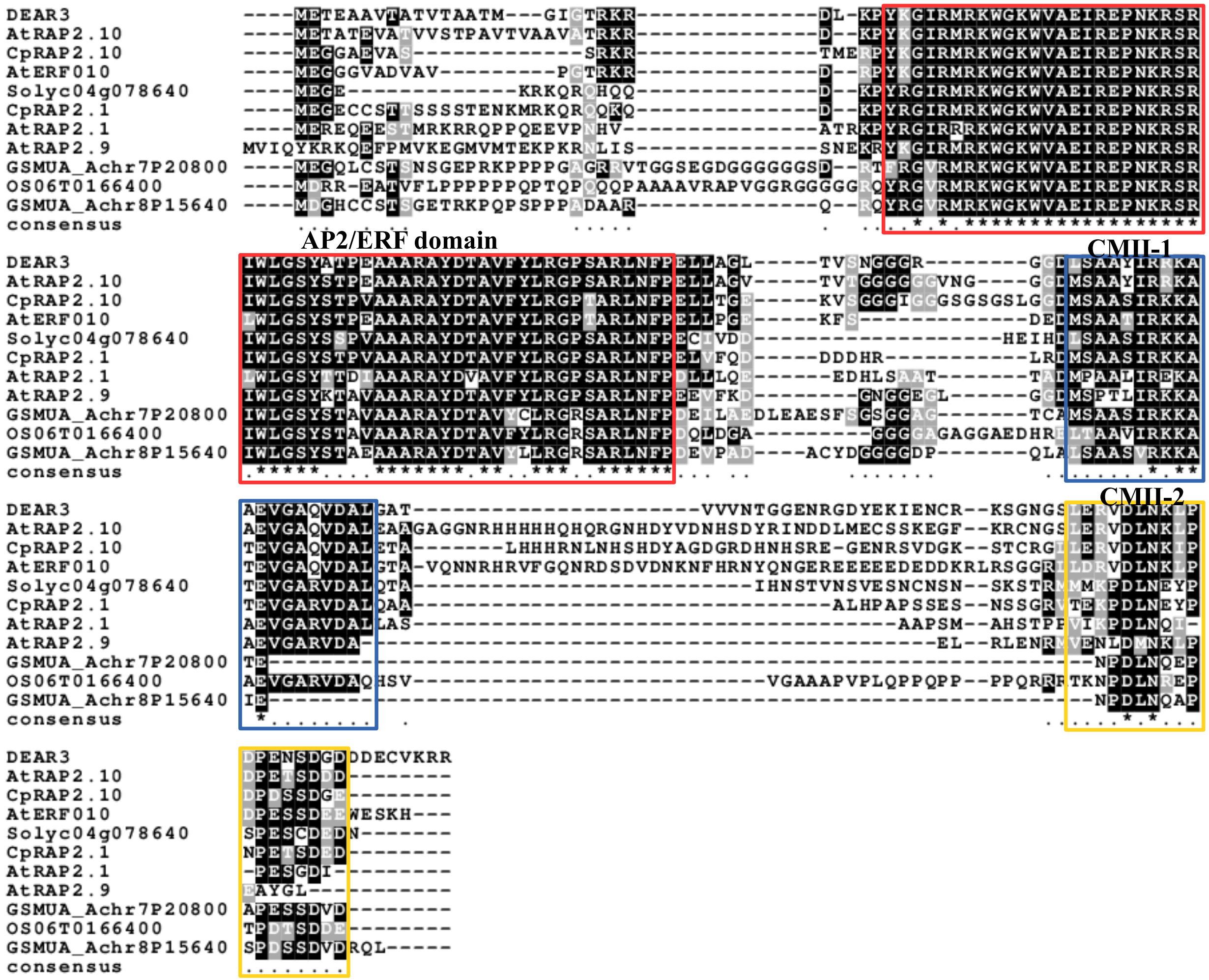

BOXSHADE analysis of the RAP2.1/2.9/2.10 subgroup belonging to group II of the AP2/ERF transcription factors.
