# Supplementary material for: RAP2.4a Is Transported through the Phloem to Regulate Cold and Heat Tolerance in Papaya Tree (Carica papaya cv. Maradol): Implications for Protection Against Abiotic Stress
Source: PLoS One. 2016 Oct 20;11(10):e0165030. doi: 10.1371/journal.pone.0165030 (PMC5072549; doi:10.1371/journal.pone.0165030)
Supplement: S3 Fig — The RNA was synthesized in vitro from the pET15b::fibrillarin vector with T7 RNA polymerase at 37°C for 1 hour. The translation was performed with a 0.08-mM amino acid mixture minus methionine, 75-mM potassium acetate, 1 μl of RNasin (40 μ/ul), 2 μl of [35S] methionine at 10 mCi/ml, 0.5 μg/ul of fibrillarin RNA and 25 μl of C. papaya sap in a total volume of 50 μl with nuclease-free water. The sap was suspended in storage buffer (24-mM HEPES buffer pH 7.4, 101-mM potassium acetate, 4.2-mM magnesium acetate, 11-mM DTT and 1-mM spermidine). The reaction was incubated at 30°C for 2 hours. The sample was heated at 100°C for two minutes to denature the proteins and then loaded in a 10% SDS-PAGE gel. The gel was allowed to dry at 80°C for 90 minutes in a vacuum chamber and then was subjected to autoradiography. B) Purification of 6His-FIBRILLARIN after in vitro translation. Reaction was loaded on 50 ul Ni-Agarose resin washed and eluted with 250 mM Imidazole. Input (I), flow through (FT), washes (w1-w4) and elution’s (E1-E2) were loaded in the indicated lanes on a 12% PAGE, and silver stain for protein detection. (PDF) (PDF) [file pone.0165030.s003.pdf]

Figure S3

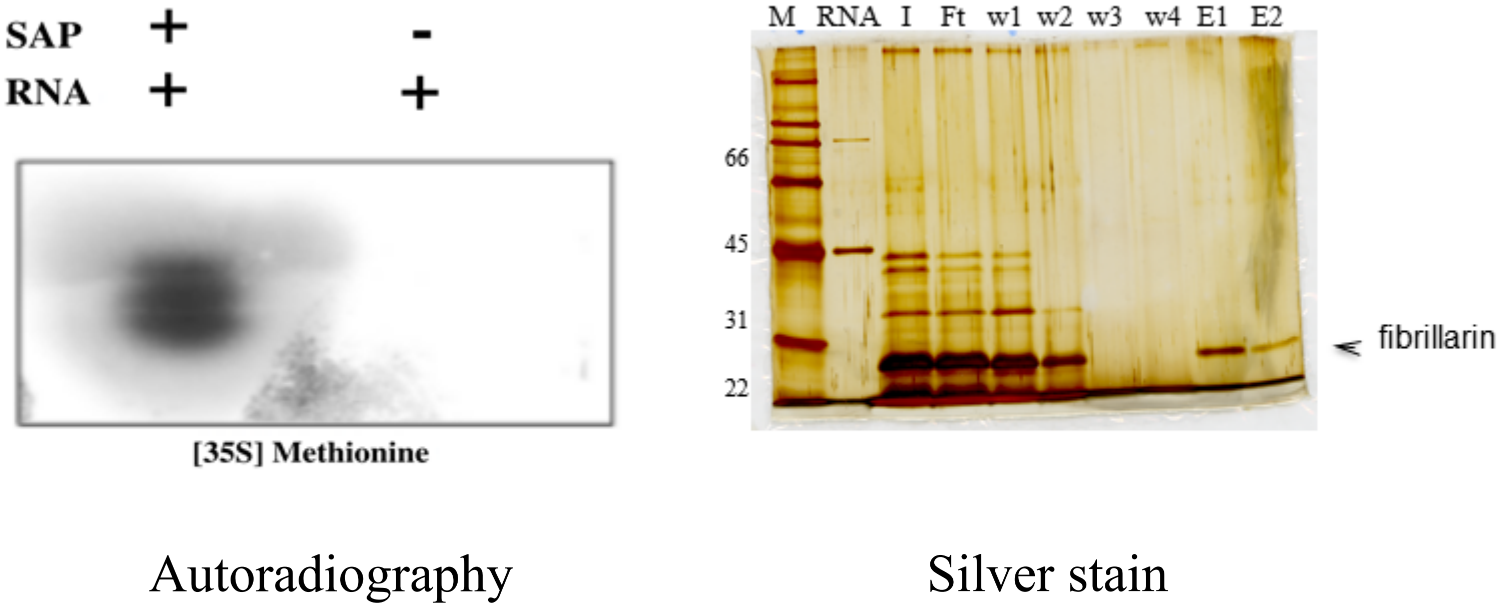

Autoradiography from an *in vitro* translation reaction with purified fibrillarin mRNA after PAGE and fibrillarin purification from an *in vitro* translation reaction.
