# Supplementary figures and images for: RAP2.4a Is Transported through the Phloem to Regulate Cold and Heat Tolerance in Papaya Tree (Carica papaya cv. Maradol): Implications for Protection Against Abiotic Stress
Source: PLoS One. 2016 Oct 20;11(10):e0165030. doi: 10.1371/journal.pone.0165030 (PMC5072549; doi:10.1371/journal.pone.0165030)

Figure S4

A) CpRAP2.4a::GFP RNA

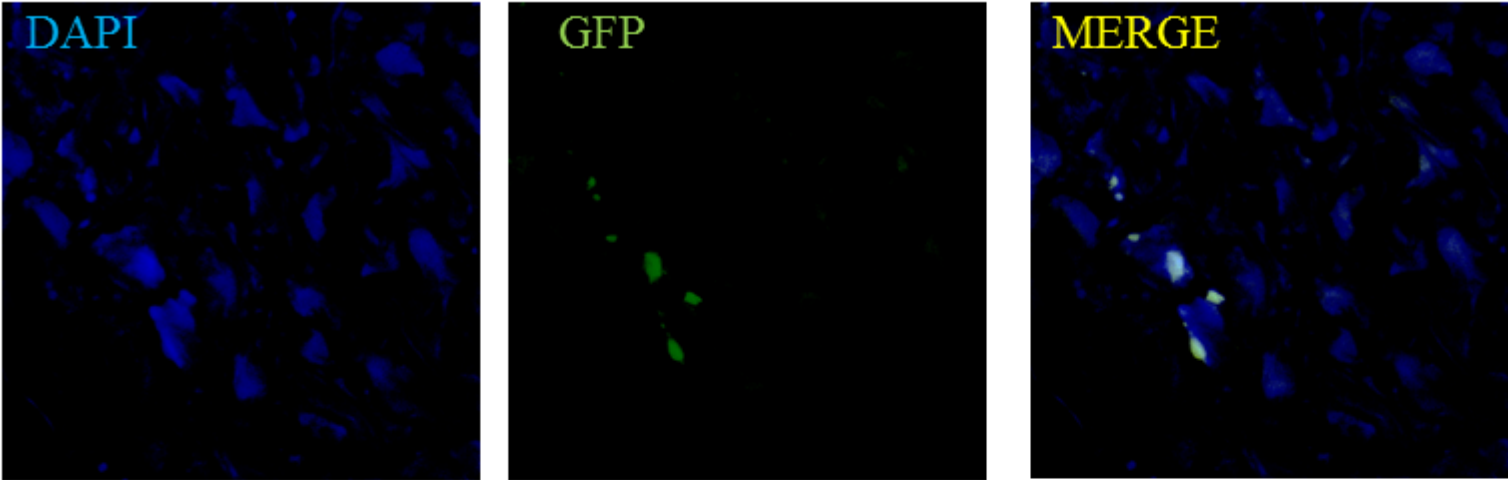

B) Control

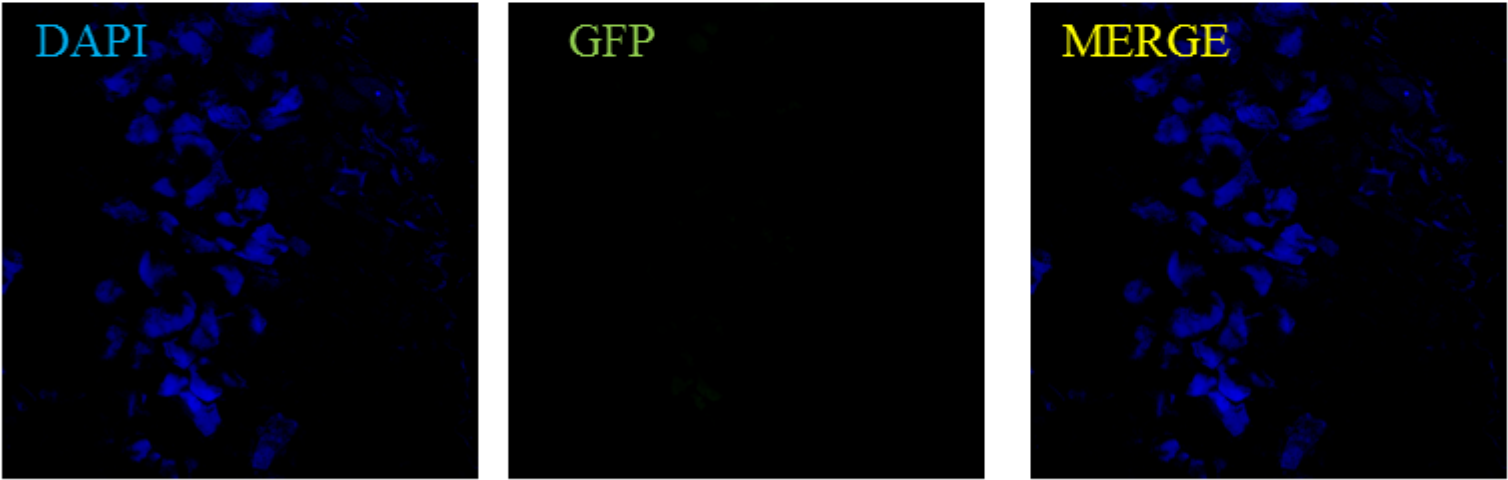

Uptake and translation of CpRAP2.4a::GFP in embryogenic callus

Supplement: S4 Fig — A) The embryogenic callus were wound 5 times with the tip of a insulin syringe that was used to add either the presence of 5 μg CpRap2.4a::gfp mRNA and then incubated for 3 days at 25°C in in MS medium containing glutamine at 0.4-mg/1 10-mg/1 2,4-D, 6% sucrose and 8-g/1 agar with the pH adjusted to 5.8. The embryos were mounted on slides with moviol with DAPI stain and analyzed the GFP fluorescence (excitation filter 488 nm, emission filter band pass of 505–530 nm). 2% of the cells showed nuclear GFP fluorescence from the embryonic tissue that was analyzed using a confocal laser-scanning microscope FV100 Olympus. DAPI staining was used to determine the location of the nuclei in cells. The experiment was carried out in triplicate (100 counted cells each time) and done on two independent times. B) Control callus without RNA incubation. (PDF) [file pone.0165030.s004.pdf]
