# Supplementary material for: RAP2.4a Is Transported through the Phloem to Regulate Cold and Heat Tolerance in Papaya Tree (Carica papaya cv. Maradol): Implications for Protection Against Abiotic Stress
Source: PLoS One. 2016 Oct 20;11(10):e0165030. doi: 10.1371/journal.pone.0165030 (PMC5072549; doi:10.1371/journal.pone.0165030)
Supplement: S5 Fig — A) MS medium without ABA, B) MS medium supplemented with ABA 1 mM. (PDF) (PDF) [file pone.0165030.s005.pdf]

Figure S5

ABA treatment

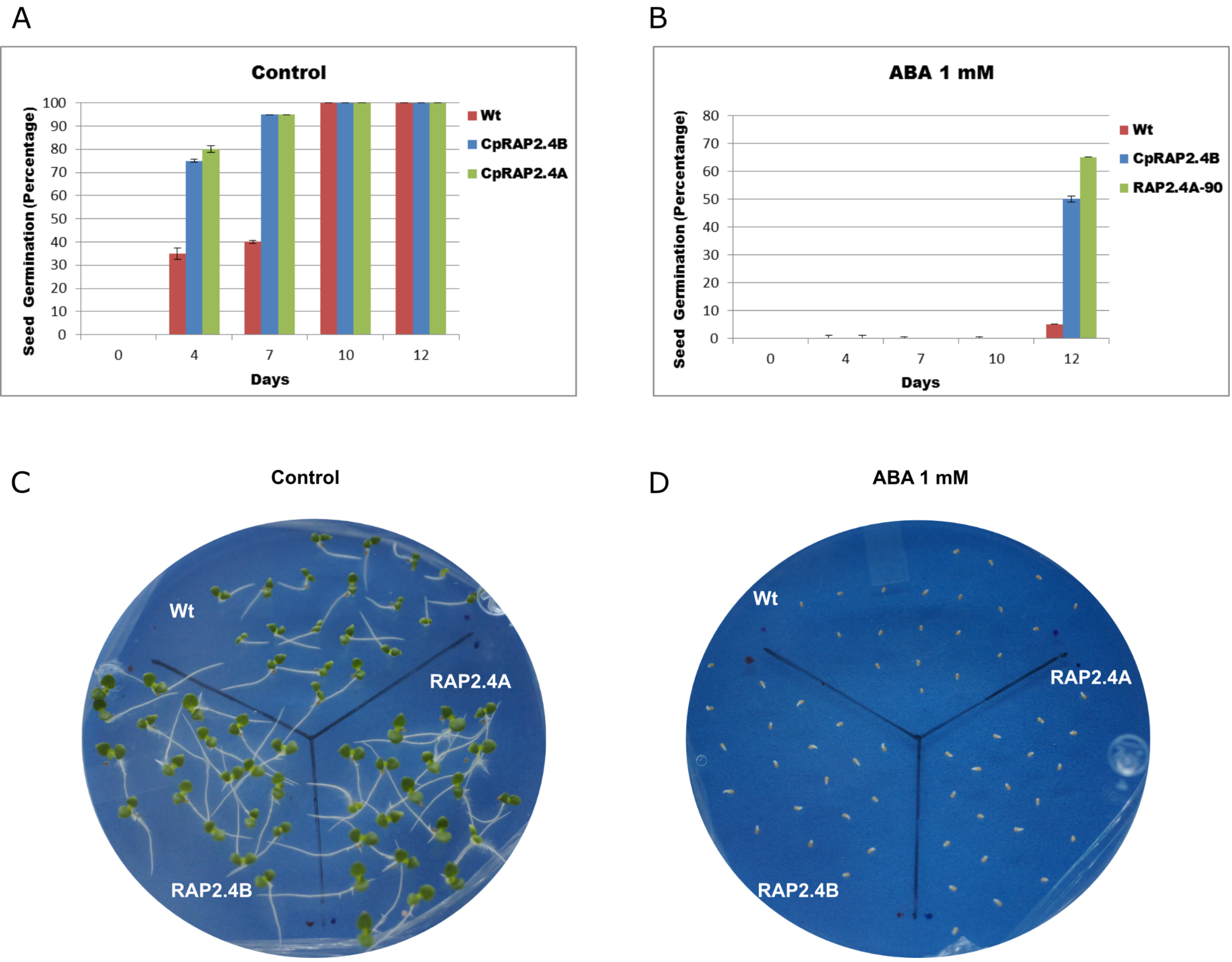

Seed germination rate of wild type, CpRAP2.4A and CpRAP2.4B overexpression lines
