# Supplementary material for: RAP2.4a Is Transported through the Phloem to Regulate Cold and Heat Tolerance in Papaya Tree (Carica papaya cv. Maradol): Implications for Protection Against Abiotic Stress
Source: PLoS One. 2016 Oct 20;11(10):e0165030. doi: 10.1371/journal.pone.0165030 (PMC5072549; doi:10.1371/journal.pone.0165030)

Figure S6

Subcellular accumulation patterns of CpRAP proteins fused to GFP in *Nicotiana tabacum* plants

A)

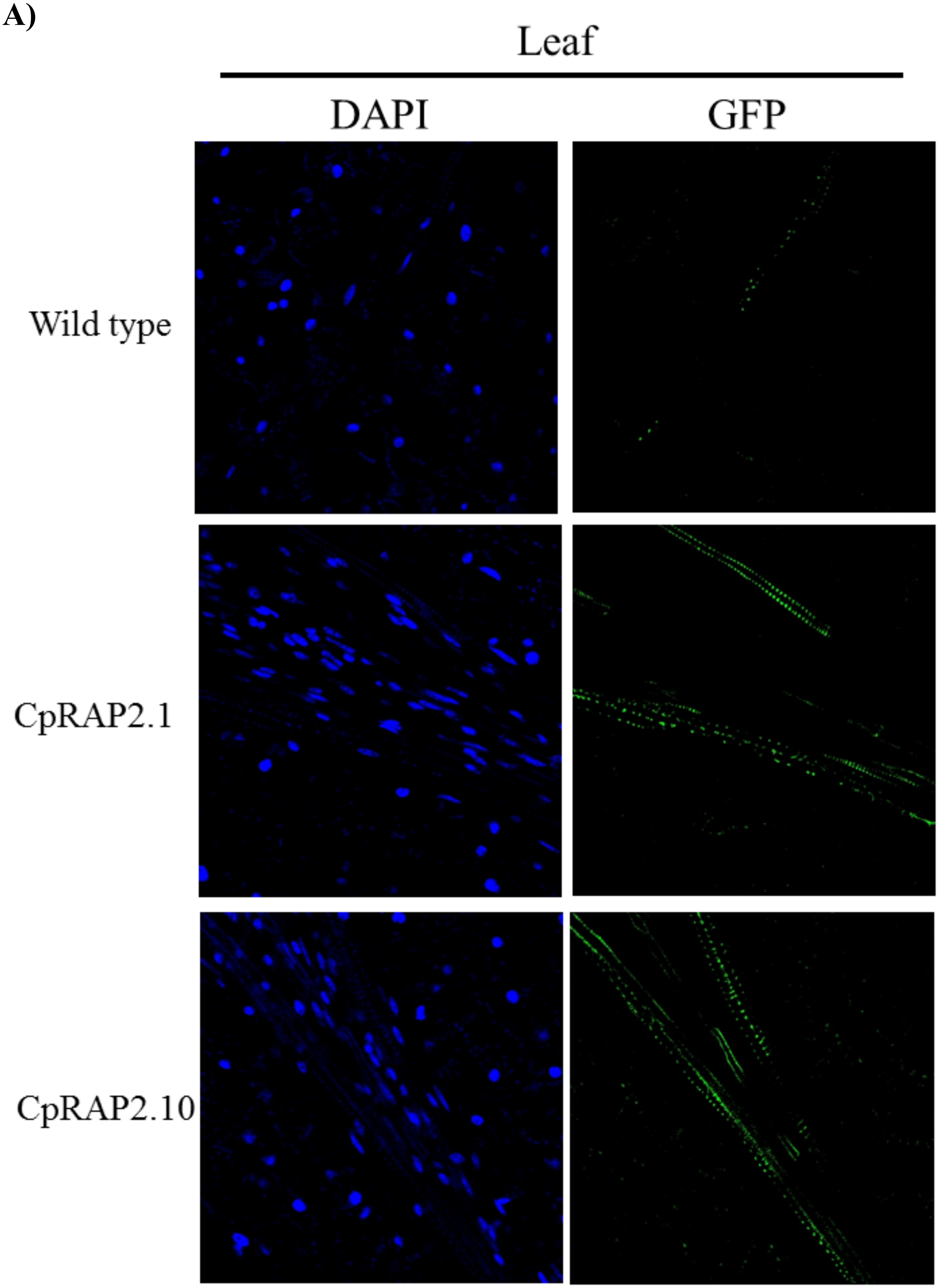

B)

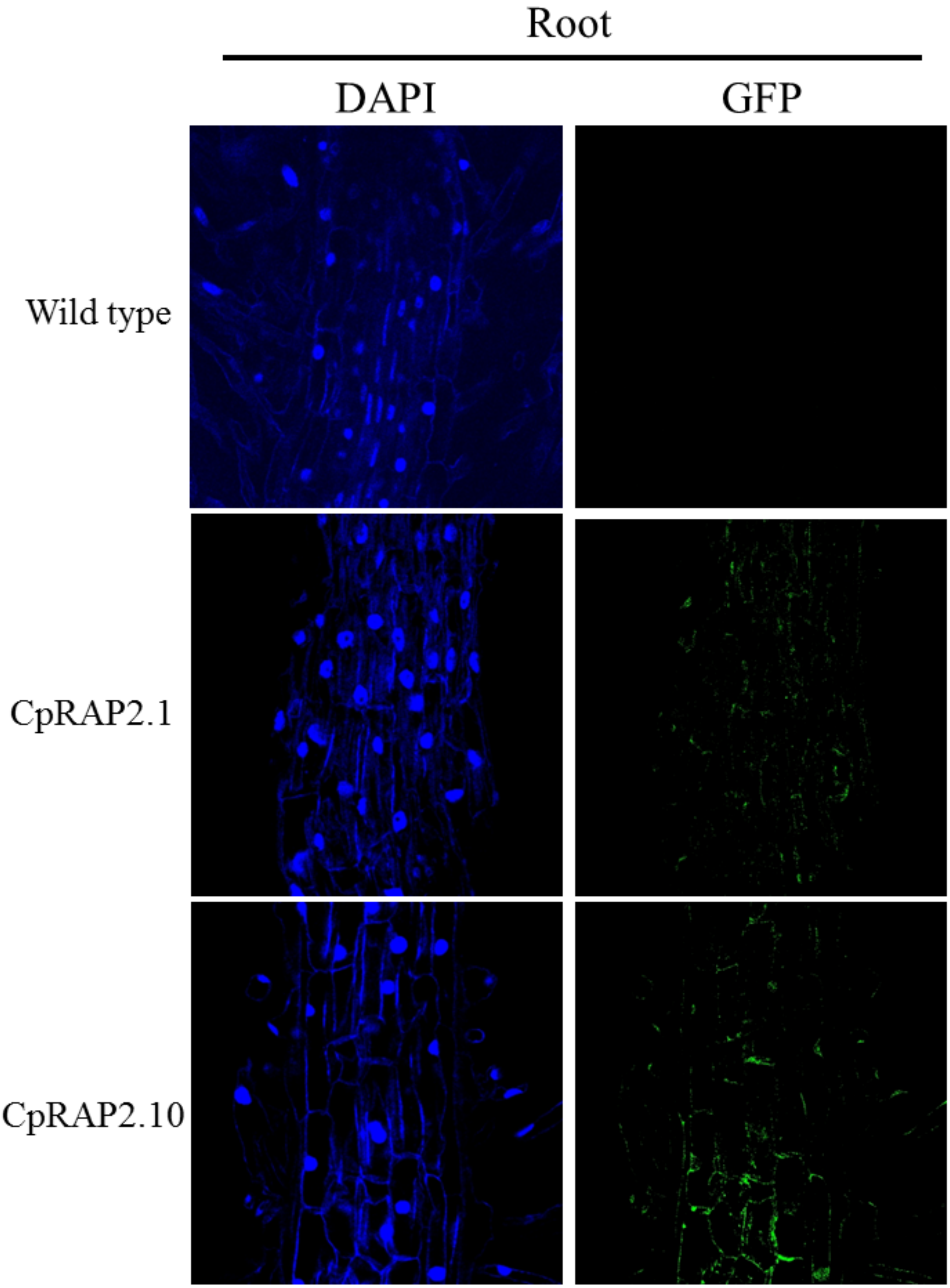

C)

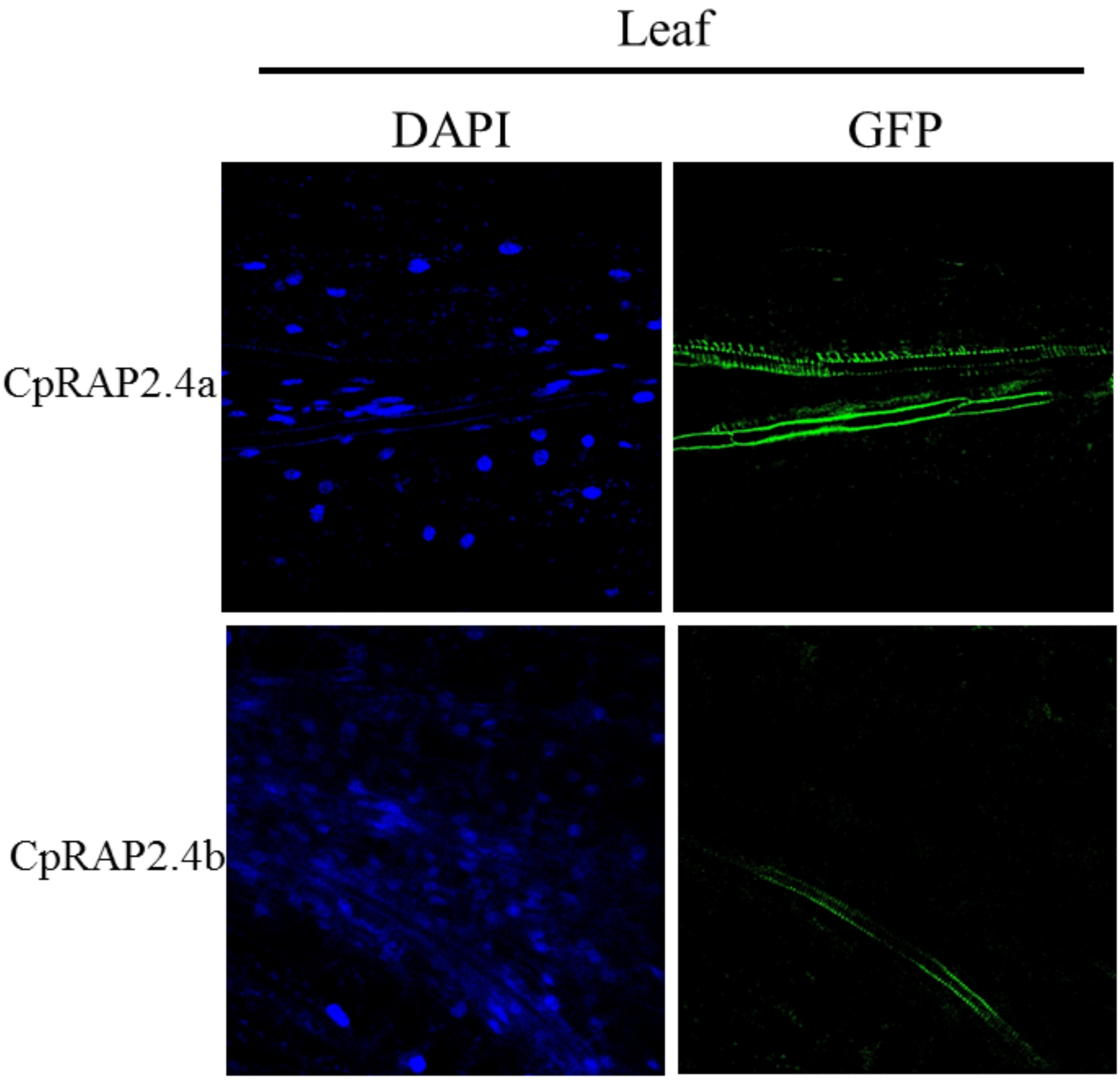

D)

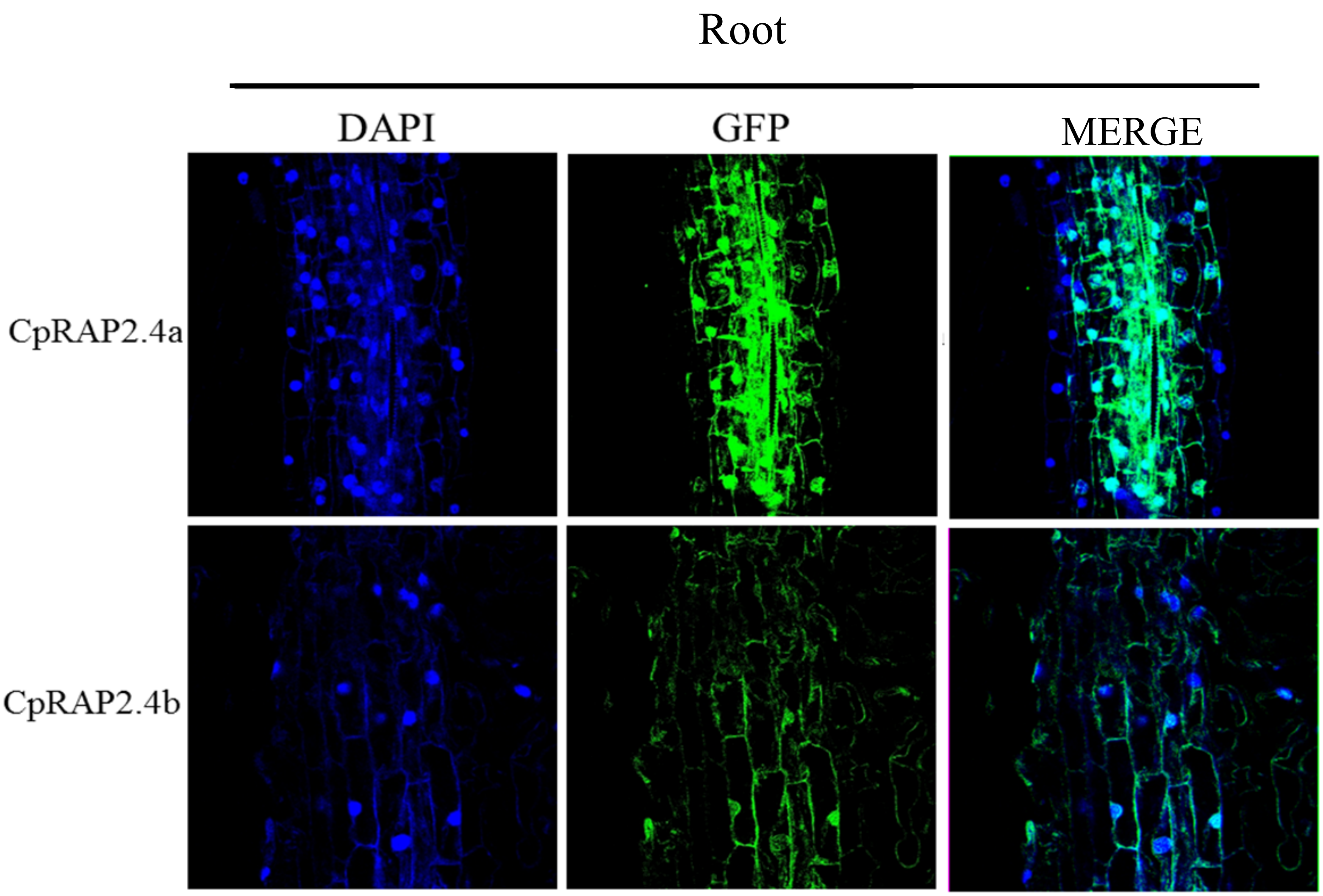

Supplement: S6 Fig — The leaves or roots of tobacco seedlings after 8 days of germination were taken under different light sources Transmitted light is observed in the horizontal first line, DAPI staining is observed in the second horizontal line and GFP is observed in the third horizontal line. DAPI staining or GFP fluorescence of leaf or root is shown for tobacco transformed plants that they carry different CpRap genes (the CpRap2.1, CpRap2.10, CpRap2.4a and CpRap2.4b gene) in their genome. The fluorescence of GFP and DAPI staining were taken at 40X on an Olympus FV1000 confocal microscope. Wild-type Tobacco plants were used as a control. A) leaf and B) root, tissues from CpRAP2.1 and CpRAP2.10 stained with DAPI. C) leaf and D) root, tissues from CpRAP2.4a and CpRAP2.4b. In S6D) merge from GFP and DAPI signals are merge to shows nuclear colocalization. (PDF) (PDF) [file pone.0165030.s006.pdf]
