# Supplementary material for: RAP2.4a Is Transported through the Phloem to Regulate Cold and Heat Tolerance in Papaya Tree (Carica papaya cv. Maradol): Implications for Protection Against Abiotic Stress
Source: PLoS One. 2016 Oct 20;11(10):e0165030. doi: 10.1371/journal.pone.0165030 (PMC5072549; doi:10.1371/journal.pone.0165030)
Supplement: S1 File — Methods for RNA expression of CpRap2.4a::gfp, micropropagation of embryogenic culture of Carica papaya, and Uptake and translation of CpRap2.4a::gfp in embryogenic callus. (PDF) (PDF) [file pone.0165030.s007.pdf]

Supplementary methods

RNA expression of CpRap2.4a::GFP

The entire open reading frames of CpRap2.4a fragments were cloned into the pDONR221 vector (Invitrogen, http://www.invitrogen.com) via the Gateway BP reaction. CpRAP2.4a (forward) 5´-AAA AAG CAG GCT TCA CCA TGC CTC AAC CTA TTT CAA ACG CG-3´ and (reverse) 5´-AGA AAG CTG GGT GTG ACA ATA TGG AGG CCC AAT CGA T-3 with the flanking attB sites into the attP sites of the pDONR™221 vector (Invitrogen, http://www.invitrogen.com). From pDONR221 clones, the fragments were recombined via a Gateway LR reaction (Invitrogen, http://www.invitrogen.com/) into the two attR recombination sites of the Gateway-compatible vector pDEST17 (Invitrogen, http://www.invitrogen.com). The *E. coli* expression strain BL21 [41] was transformed with the expression vector pDEST17-CpRAP2.4a. Recombinant protein synthesis was induced upon the addition of isopropyl β-d-thiogalactopyranoside (1-mM final concentration) during exponential growth at OD600 = 0.4 for 4 h at 37 °C. The recombinant protein CpRAP2.4a::GFP was purified via His-tag using Ni2+-NTA beads (QIAGEN, https://www.qiagen.com) under denaturing conditions. To verified that the bacteria were expressing the RNA for CpRAP2.4::GFP. Total RNA was extracted from the same batch of cells using the TRIzol Reagent (Ambion, http://www.ambion.com). Residual DNA present in RNA preparations following purification was removed using a RNase-free DNase I (New England Biolabs, https://www.neb.com) treatment according to the manufacturer's instructions.

Micropropagation of embryogenic culture of Carica papaya

Embryos were cultured for embryogenic callus induction and propagation at 26 °C under darkness in A10 medium consisting of half strength macronutrients and micronutrients, iron, myo-inositol and vitamins of MS medium [37] plus glutamine at 0.4-mg/1, 10-mg/1 2,4-D, 6% sucrose and 8-g/1 agar with the pH adjusted to 5.8[38].

Uptake and translation of CpRap2.4a::GFP in embryogenic callus.

The embryogenic callus were wound 5 times with the tip of a insulin syringe that was used to add either the presence of 5 μg CpRAP2.4a::GFP mRNA and then incubated for 3 days at 25 °C in in MS medium containing glutamine at 0.4-mg/1 10-mg/1 2,4-D, 6% sucrose and 8-g/1 agar with the pH adjusted to 5.8. The embryos were mounted on slides with mowiol with DAPI stain and analized the GFP fluorescence (excitation filter 488 nm, emission filter band pass of 505-530 nm). 2% of the cells showed nuclear GFP fluorescence from the embryonic tissue that was analyzed using a confocal laser-scanning microscope FV100 Olympus. DAPI staining was used to determine the location of the nuclei in cells. The experiment was carried out in triplicate (100 counted cells each time) and done on two independent times.
