# Supplementary material for: RAP2.4a Is Transported through the Phloem to Regulate Cold and Heat Tolerance in Papaya Tree (Carica papaya cv. Maradol): Implications for Protection Against Abiotic Stress
Source: PLoS One. 2016 Oct 20;11(10):e0165030. doi: 10.1371/journal.pone.0165030 (PMC5072549; doi:10.1371/journal.pone.0165030)
Supplement: S1 Table — We identified and retrieved 80 sequences with the AP2/ERF domain from C. papaya tree and that were named CpERFs. The groups were clustered according to Nakano et al. (2006). (PDF) (PDF) [file pone.0165030.s008.pdf]

S1 Table. List of the AP2/ERF TF family in Carica papaya.

Name	C. papaya ID taq	Group	Best BLAST and GenBank accession No.	E value
CpERF1	evm.model.supercontig_72.47	X	PREDICTED: ethylene-responsive transcription factor ABR1 [*Cucumis melo*], XP_008440983.1	3e-48
CpERF2	evm.model.supercontig_38.79	X	AP2 6l, putative [*Theobroma cacao*], XP_007043782.1	2E-057
CpERF3	evm.model.supercontig_49.94	X	AP2 domain-containing transcription factor family protein [*Populus trichocarpa*], XP_002306712.2	6E-098
CpERF4	evm.model.supercontig_83.80	VIII	AP2/ERF super family protein [*Hevea brasiliensis*], AFX83939.1	2E-060
CpERF5	evm.model.supercontig_89.73	III	Integrase-type DNA-binding superfamily protein, putative [*Theobroma cacao*], XP_007039780.1	2E-068
CpERF6	evm.model.supercontig_2304.1	VII	AP2/EREBP transcription factor ERF-2 [*Gossypium hirsutum*], AAV51937.1	4E-075
CpERF7	evm.model.supercontig_62.11	X	hypothetical protein JCGZ_01609 [*Jatropha curcas*], KDP42285.1	4E-075
CpERF8	evm.model.supercontig_131.83	VIII	ATERF-4/ATERF4/ERF4/RAP2.5 [*Arabidopsis lyrata* subsp. Lyrata], XP_002882920.1	5E-055
CpERF9	evm.model.supercontig_101.15	IX	Ethylene-responsive element binding factor 13, putative [*Theobroma cacao*], XP_007052304.1	2E-051
CpERF10	evm.model.supercontig_27.18	VIII	PREDICTED: ethylene-responsive transcription factor 12-like [*Vitis vinifera*], XP_002279760.1	5E-070
CpERF11	evm.model.supercontig_13.175	X	unnamed protein product [*Vitis vinifera*], CBI15759.*3*	1E-054
CpERF12	evm.model.supercontig_27.16	VIII	unnamed protein product [*Vitis vinifera*], CBI30599.3	1E-059
CpERF13	evm.model.supercontig_101.39	III	PREDICTED: ethylene-responsive transcription factor ERF034-like [*Cicer arietinum*], XP_004510113.1	1E-054
CpERF14	evm.model.supercontig_89.42	IX	Ethylene response factor 5, putative [*Theobroma cacao*], XP_007039738.1	2E-093
CpERF15	evm.model.supercontig_21.69	III	PREDICTED: ethylene-responsive transcription factor ERF021 [*Cucumis melo*], XP_008459604.1	3E-075
CpERF16	evm.model.supercontig_44.73	IX	PREDICTED: ethylene-responsive transcription factor ERF098 [*Prunus mume*], XP_008224367.1	7E-058
CpERF17	evm.model.supercontig_12.251	VIII	C-repeat binding transcription factor 2 [*Jatropha curcas*], AIC83902.1	3E-085
CpERF18	evm.model.supercontig_40.39	III	hypothetical protein JCGZ_07297 [*Jatropha curcas*], KDP33726.1	1E-056
CpERF19	evm.model.supercontig_481.1	VII	Transcription factor JERF1, putative isoform 1 [*Theobroma cacao*], XP_007024746.1	2E-095
CpERF20	evm.model.supercontig_21.70	III	AP2 domain-containing transcription factor family protein [*Populus trichocarpa*],XP_002325520.2	2E-067
CpERF21	evm.model.supercontig_132.31	VIII	ethylene response factor ERF4 [*Solanum tuberosum*], AEM63545.1	5E-063
CpERF22	evm.model.supercontig_7.269	III	Integrase-type DNA-binding superfamily protein [*Theobroma cacao*], XP_007012129.1	2E-068
CpERF23	evm.model.supercontig_10.113	V	DNA binding protein, putative [*Ricinus communis*], XP_002527321.1	9E-078
CpERF24	evm.model.supercontig_50.28	IX	hypothetical protein JCGZ_17701 [*Jatropha curcas*], KDP26543.1	1E-046
CpERF25	evm.model.supercontig_2.277	VI	PREDICTED: pathogenesis-related genes transcriptional activator PTI6-like [*Prunus mume*], XP_008240070.1	5E-068
CpERF26	evm.model.supercontig_13.250	IX	ethylene responsive element binding factor-like [*Carica papaya*], AGT21474.1	0
CpERF27	evm.model.supercontig_194.13	VI	AP2/ERF domain-containing transcription factor, putative [*Theobroma cacao],* XP_007051632.1	2E-088
CpERF28	evm.model.supercontig_34.132	I	ethylene-responsive transcriptionfactor 4 [*Carica papaya*], AFV60736.1	0
CpERF29	evm.model.supercontig_7.36	IX	hypothetical protein POPTR_0004s05110g [*Populus trichocarpa*],	2E-089

			XP_002305098.1	
CpERF30	evm.model.supercontig_50.29	IX	PREDICTED: ethylene-responsive transcription factor ERF098-like [*Cicer arietinum*], XP_004493770.1	2E-031
CpERF31 / CpRAP2.4a	evm.model.supercontig_36.112	I	ethylene-responsive element binding-factor [*Jatropha curcas*], AFY98895.1	9E-133
CpERF32	evm.model.supercontig_109.32	III	hypothetical protein CICLE_v10027587mg [*Citrus clementina*], XP_006426907.1	3E-062
CpERF33 / CpRAP2.4b	evm.model.supercontig_996.1	I	AP2/ERF domain-containing transcription factor, putative [T*heobroma cacao*], XP_007047994.1	3E-133
CpERF34	evm.model.supercontig_151.30	VII	hypothetical protein PRUPE_ppa008730mg [*Prunus persica*], XP_007200418.1	2E-103
CpERF35	evm.model.supercontig_55.155	X	PREDICTED: ethylene-responsive transcription factor ERF109 [*Prunus mume*], XP_008236506.1	4E-032
CpERF36	evm.model.supercontig_44.74	IX	hypothetical protein PRUPE_ppa018601mg [*Prunus persica*], XP_007224270.1	3E-043
CpERF37 / CpDREB_IV3	evm.model.supercontig_99.20	IV	Dehydration-responsive element-binding protein 2G, putative [*Theobroma cacao*], XP_007017661.1	6E-078
CpERF38	evm.model.supercontig_3.43	VI	Ethylene-responsive transcription factor, putative [*Theobroma cacao*], XP_007038942.1	1E-120
CpERF39	evm.model.supercontig_70.47	VIII	hypothetical protein JCGZ_26896 [*Jatropha curcas*], KDP41878.1	4E-068
CpERF40 / CpDREB_IV5	evm.model.supercontig_1288.2	IV	hypothetical protein CISIN_1g038194mg [*Citrus sinensis*], KDO68917.1	2E-071
CpERF41	evm.model.supercontig_162.25	IX	hypothetical protein PRUPE_ppa016384mg [*Prunus persica*], XP_007206287.1	1E-073
CpERF42	evm.model.supercontig_122.27	VI	Cytokinin response factor 2, putative [*Theobroma cacao*], XP_007040431.1	5E-101
CpERF43	evm.model.supercontig_127.4	V	PREDICTED: ethylene-responsive transcription factor ERF003-like [*Malus domestica*], XP_008377674.1	1E-087
CpERF44	evm.model.supercontig_44.71	IX	Ethylene-responsive transcription factor 1B [*Theobroma cacao*], XP_007035093.1	3E-094
CpERF45	evm.model.supercontig_79.43	I	hypothetical protein JCGZ_20463 [*Jatropha curcas*], KDP25307.1	3E-122
CpERF46	evm.model.supercontig_10.254	V	hypothetical protein CICLE_v10027305mg [*Citrus clementina*], XP_006427266.1	3E-080
CpERF47	evm.model.supercontig_266.5	II	ethylene-responsive transcription factor 2 [*Carica papaya*], AFV60734.1	2E-155
CpERF48	evm.model.supercontig_13.253	IX	ethylene-responsive transcription factor 3 [*Carica papaya*], AFV60735.1	5E-146
CpERF49	evm.model.supercontig_33.29	VIII	DORNROSCHEN-like, putative [*Theobroma cacao*], XP_007044810.1	2E-089
CpERF50	evm.model.supercontig_10.125	VIII	PREDICTED: uncharacterized protein LOC101314304 [*Fragaria vesca* subsp. Vesca], XP_004305488.1	8E-072
CpERF51	evm.model.supercontig_101.16	IX	hypothetical protein JCGZ_14625 [*Jatropha curcas*], KDP28854.1	1E-040
CpERF52	evm.model.supercontig_5.71	III	PREDICTED: ethylene-responsive transcription factor ERF024 [*Vitis vinifera*], XP_002283401.2	2E-075
CpERF53 / CpDREB_IV4	evm.model.supercontig_92.16	IV	Integrase-type DNA-binding superfamily protein [*Theobroma cacao*], XP_007028832.1	3E-141
CpERF54	evm.model.supercontig_18.194	VIII	AP2/ERF domain-containingtranscription factor [*Theobroma cacao*], XP_007051933.1	2E-106
CpERF55	evm.model.supercontig_74.84	III	Dehydration-responsive element-binding protein 1F, putative [R*icinus communis*], XP_002509703.1	4E-093
CpERF56 / CpDREB_IV2	evm.model.supercontig_51.23	IV	hypothetical protein CICLE_v10022244mg [*Citrus clementina*], XP_006443285.1	3E-053
CpERF57	evm.model.supercontig_51.136	VIII	PREDICTED: ethylene-responsive transcription factor RAP2-11 [*Vitis vinifera*], XP_002281619.1	1E-105

CpERF58	evm.model.supercontig_16.148	VIII	hypothetical protein VITISV_003244 [*Vitis vinifera*], CAN78552.1	3E-078
CpERF59	evm.model.supercontig_259.2	V	PREDICTED: ethylene-responsive transcription factor SHINE 3-like [Vitis vinifera], XP_002285107.1	2E-076
CpERF60	evm.model.supercontig_62.63	VIII	AP2/ERF domain-containing transcription factor, putative [T*heobroma cacao*], XP_007039294.1	7E-049
CpERF61 / CpDREB_IV1	evm.model.supercontig_19.121	IV	Dehydration-responsive element-binding protein 2C, putative [*Ricinus communis*], XP_002520794.1	6E-110
CpERF62	evm.model.supercontig_151.29	VII	Ethylene responsive transcription factor 2b [*Medicago truncatula*], XP_003591474.1	4E-058
CpERF63	evm.model.supercontig_14.126	VIII	WIN1-like protein, putative [*Theobroma cacao*], XP_007032619.1	8E-125
CpERF64	evm.model.supercontig_36.41	II	AP2 domain-containing transcription factor family protein [P*opulus trichocarpa*], XP_006386376.1	9E-068
CpERF65	evm.model.supercontig_5.66	III	CBF/DREB1 transcription factor 1 [*Carica papaya*], AFV15809.1	3E-127e
CpERF66	evm.model.supercontig_91.71	II	ethylene-responsive transcription factor ERF015 [*Arabidopsis thaliana*], NP_567867.1	3E-069
CpERF67	evm.model.supercontig_74.86	III	ethylene-responsive transcription factor 1 [*Carica papaya*], AFV60733.1	5E-153
CpERF68	evm.model.supercontig_13.83	VIII	hypothetical protein RCOM_1618730 [*Ricinus communis*], XP_002512003.1	1E-048
CpERF69	evm.model.supercontig_151.27	VII	uncharacterized protein LOC100780763 [*Glycine max*], NP_001241446.1	2E-069
CpERF70	evm.model.supercontig_21.63	II	Integrase-type DNA-binding superfamily protein, putative [*Theobroma cacao*], XP_007019844.1	4E-051
CpERF71 / CpDREB_IV6	evm.model.supercontig_5.232	IV	hypothetical protein PHAVU_008G092800g [*Phaseolus vulgaris*], XP_007140202.1	3E-045
CpERF72	evm.model.supercontig_71.10	I	c-repeat binding transcription factor 2 [*Jatropha curcas*], AIC83902.1	2E-042
CpERF73	evm.model.supercontig_1.178	X	Integrase-type DNA-binding superfamily protein, putative isoform 1 [*Theobroma cacao*], XP_007046241.1	2E-092
CpERF74	evm.model.supercontig_20.67	III	CRT/DRE binding factor 1 [*Morus alba* var. Multicaulis], AFQ59977.1	2E-080
CpERF75	evm.model.supercontig_7.130	VI	hypothetical protein POPTR_0002s24750g [*Populus trichocarpa*], XP_006386876.1	3E-100
CpERF76	evm.model.supercontig_21.106	XI	hypothetical protein POPTR_0002s24750g [*Populus trichocarpa*], XP_006386876.1	3E-100
CpERF77	evm.model.supercontig_7.129	VI	Integrase-type DNA-binding superfamily protein [*Theobroma cacao*], XP_007028893.1	4E-035
CpERF78	evm.model.supercontig_224.4	XI	hypothetical protein JCGZ_21099 [*Jatropha curcas*], KDP26066.1	0
CpERF79 / CpRAP2.1	Supercontig_21 1671823-1672296	II	ERF/AP2 domain-containing transcription factor [*Manihot esculenta*], AGC51783.1	2E-073
CpERF80 / CpRAP2.10	Supercontig_55 674943-675488	II	PREDICTED: ethylene-responsive transcription factor RAP2-10-like [*Brassica rapa*], CDY62224.1	1E-065
