# Supplementary material for: RAP2.4a Is Transported through the Phloem to Regulate Cold and Heat Tolerance in Papaya Tree (Carica papaya cv. Maradol): Implications for Protection Against Abiotic Stress
Source: PLoS One. 2016 Oct 20;11(10):e0165030. doi: 10.1371/journal.pone.0165030 (PMC5072549; doi:10.1371/journal.pone.0165030)
Supplement: S2 Table — Each band was quantified and normalizes by dividing the value from the 0 time and then multiplying the value by 100. (PDF) (PDF) [file pone.0165030.s009.pdf]

Table S2

Normalized value of RT-PCR from Figure 2

		4º C						40 º C		
100	52	57	185	253		100	73	554	447	139
100	153	304	239	184		100	368	424	490	216
100	162	233	293	236		100	164	271	501	563
100	143	171	164	155		100	114	125	122	138
100	1337	1237	1622	1757		100	384	443	343	244
100	131	154	150	173		100	319	248	286	229
100	101	105	98	105		100	116	121	125	124
100	940	757	1507	1121		100	253	950	1219	77
100	275	149	161	193		100	382	370	372	280
100	235	204	245	161		100	215	296	525	1063
100	902	916	925	530		100	590	614	713	642
100	512	355	424	401		100	1012	978	1081	880
100	154	138	140	57		100	214	201	225	272
100	103	105	107	108		100	79	85	85	82
100	48	108	108	129		100	121	104	109	121
100	198	188	216	155		100	418	369	345	176
100	116	143	135	134		100	103	119	158	192
100	155	141	160	167		100	168	155	161	148
100	183	136	166	170		100	168	169	141	184
100	185	165	176	189		100	326	259	295	248
100	108	113	117	110		100	120	121	122	126
