# Supplementary material for: RAP2.4a Is Transported through the Phloem to Regulate Cold and Heat Tolerance in Papaya Tree (Carica papaya cv. Maradol): Implications for Protection Against Abiotic Stress
Source: PLoS One. 2016 Oct 20;11(10):e0165030. doi: 10.1371/journal.pone.0165030 (PMC5072549; doi:10.1371/journal.pone.0165030)
Supplement: S3 Table — Each band was quantified and normalizes by dividing the value from the 0 time and then multiplying the value by 100. (PDF) (PDF) [file pone.0165030.s010.pdf]

Table S3

Normalized value of RT-PCR from Figure 3

whole plant			sap		
C	40ºC	4ºC	C	40ºC	4ºC
100	397	584	-	-	-
100	118	131	100	107	123
100	208	204	100	223	208
100	175	127	100	311	555
100	179	162	-	100	90
100	167	146	100	124	124
100	117	117	100	102	97
